# Single‐residue posttranslational modification sites at the N‐terminus, C‐terminus or in‐between: To be or not to be exposed for enzyme access

**DOI:** 10.1002/pmic.201400633

**Published:** 2015-07-14

**Authors:** Fernanda L. Sirota, Sebastian Maurer‐Stroh, Birgit Eisenhaber, Frank Eisenhaber

**Affiliations:** ^1^ Bioinformatics Institute (BII)Agency for Science and Technology (A*STAR)MatrixSingapore; ^2^ School of Biological Sciences (SBS)Nanyang Technological University (NTU)Singapore; ^3^ Department of Biological Sciences (DBS)National University of Singapore (NUS)Singapore; ^4^ School of Computer Engineering (SCE)Nanyang Technological University (NTU)Singapore

**Keywords:** Accessibility, Bioinformatics, Posttranslational modifications, Protein, Structure

## Abstract

Many protein posttranslational modifications (PTMs) are the result of an enzymatic reaction. The modifying enzyme has to recognize the substrate protein's sequence motif containing the residue(s) to be modified; thus, the enzyme's catalytic cleft engulfs these residue(s) and the respective sequence environment. This residue accessibility condition principally limits the range where enzymatic PTMs can occur in the protein sequence. Non‐globular, flexible, intrinsically disordered segments or large loops/accessible long side chains should be preferred whereas residues buried in the core of structures should be void of what we call canonical, enzyme‐generated PTMs. We investigate whether PTM sites annotated in UniProtKB (with MOD_RES/LIPID keys) are situated within sequence ranges that can be mapped to known 3D structures. We find that N‐ or C‐termini harbor essentially exclusively canonical PTMs. We also find that the overwhelming majority of all other PTMs are also canonical though, later in the protein's life cycle, the PTM sites can become buried due to complex formation. Among the remaining cases, some can be explained (i) with autocatalysis, (ii) with modification before folding or after temporary unfolding, or (iii) as products of interaction with small, diffusible reactants. Others require further research how these PTMs are mechanistically generated in vivo.

## 1 Introduction

Covalent chemical alterations of a protein's primary structure, also known as protein PTMs are important events in the maturation process of a polypeptide chain. PTMs can take place at any time point in the life time of a protein posttranslationally but also co‐translationally during the protein biosynthesis process. N‐terminal glycine N‐myristoylation is an example of a PTM that can occur either way 1, 2.

For an alteration to be considered a typical PTM, one would expect it to be observed among proteins from different families, not just in a group of sequentially very similar ones. With PTMs, proteins go beyond the limitations (i) in the genome‐encoded primary sequences and (ii) in the set of just 20 natural amino acid monomers. From the chemical point of view, PTMs involve both the formation of covalent links of intra‐ or intermolecular (with a ligand group/another protein) nature and the cleavage of covalent bonds including the breakage of a peptide bond and the removal of groups from single amino acid types. PTMs greatly influence protein size, hydrophobicity, charge and other physico‐chemical properties and, therefore, change/enhance/inhibit specific protein activities or target the protein to another subcellular localization. Thus, PTMs directly modify proteins’ molecular functions.

**Figure 1:**
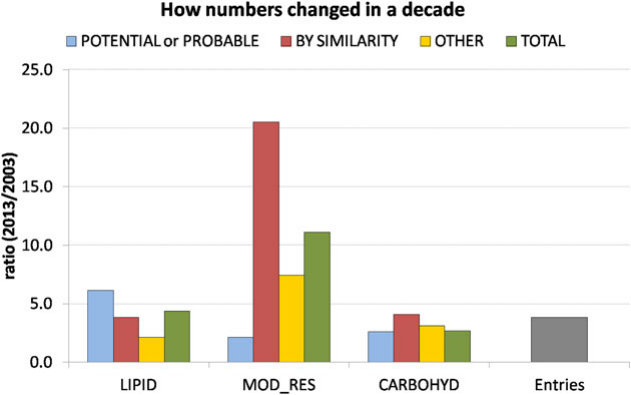
The ratio between the numbers of PTMs and entries calculated for a 10 year period. The numbers of PTMs were additionally split according to their annotation in UniProtKB/Swiss‐Prot: potential or probable in blue, by similarity in red, other in yellow (likely to be experimentally verified) and total in green, representing the sum of all three. The ratio for the numbers of entries is in gray.

In classical biochemistry courses presented to the current generation of team leaders during their time as students, PTMs of proteins were dealt with only marginally and the impression might have arisen that a PTM is a rare, extraordinary event. As we know today, nothing is farther from the truth. In the age of proteomics, PTMs are no longer an obscure phenomenon. Typically, a protein is multiply covalently modified during its life cycle, thereby experiencing changes of its functional state 3. The UniProtKB feature table (FT) lines with the tokens “MOD_RES”, “LIPID”, etc. provide a convenient handle to assess the status of proteins’ PTM annotations 4. The number of PTM instances has already been larger than the number of protein sequence entries in databases at the time of our 2003 review 3 and this trend has continued during the last decade (Fig. 1). Whereas the number of protein entries in UniProtKB 4 has grown ∼3.8 fold, the number of annotated lipidation cases increased >4 fold and that of MOD_RES annotated instances even ∼11 fold. In the same period, the diversity of LIPID annotation types in the FT has grown from 26 to 38 and single‐residue chemical modifications of the MOD_RES type have increased from 45 to 275. Only the number of annotated glycosylation examples remains below the sequence growth (increased by a factor of 2.7), most likely due to continued experimental difficulties in their large‐scale determination and the absence of progress in glycosylation site prediction 5. To note, the relative abundance of experimentally verified instances of various PTMs is unlikely to correlate with their frequency in nature. In contrast, it is rather biased by the difficulty of the respective experimental procedures. Since the high‐throughput techniques for large‐scale PTM determination are still in their infancy for many PTM types, discovery of new protein PTMs should not be considered a surprise and some PTMs, which are considered an oddity today, might receive fundamental importance in the future. Thus, both the proteome 6 as well as the list of PTMs occurring in proteomes of living systems are apparently far from complete today.

In this work, we wish to explore the relationship between PTMs that exclusively affect the chemical structure of a single residue and their location in the protein sequence with regard to regions having globular, tertiary structure as well as to various non‐globular segments 3, 5. In this context, it is important to realize that typical PTMs originate from the action of posttranslationally modifying enzymes. These PTMs represent a regulated information transfer event in pathways and, thus, are of special biological significance. In contrast, most but apparently not all PTMs that spontaneously occur, for example, as a result of long‐term exposure to pathologically increased concentrations of low‐molecular reactants 7, 8, 9 are not part of the normal physiology. Whereas, in the latter case, a small‐volume metabolite or xenobiotic compound can attack the polypeptide chain essentially anywhere (to the extent that, within long time frames, rare conformational fluctuations allow access to internal residues of tertiary structures), generally, posttranslationally modifying enzymes have an access problem 10. The respective PTMs are introduced only into substrate proteins that carry a sequence motif that is recognized by the posttranslationally modifying enzymes. This means that the motif carrying sequence segment from the substrate protein has to enter the catalytic crevice in the 3D structure of the modifying enzyme. Only sites that fulfill this accessibility condition can receive the respective PTM 10, 11.

The expected size of the accessible segment carrying the enzyme‐generated PTM site has been estimated in posttranslational modification and translocation signal predictor developments 12, 13, 14, 15, 16, 17. Regardless of the specific nature of the PTM, the general architecture of the sequence motif is common 3, 10, 11. A small central segment (typically, about five residues) that is specific for the PTM and that directly enters the catalytic cleft of the enzyme is surrounded by segments with linker characteristics on either side (with ≥10 residues that, as a trend, are polar for interaction with the aqueous environment, of small volume and with flexible backbone). It is the linker surrounding that makes the PTM site mechanically accessible to the enzyme's catalytic site.

Thus, the accessibility condition requires the PTM site to be located in a flexible region, without strong 3D structural preferences, so that the respective sequence segment of the substrate protein can conformationally adapt to the geometry of the binding site of the posttranslationally modifying enzyme. These conditions are more likely to be met in non‐globular sections of the sequence 10, for example in intrinsically unfolded segments 18, 19, 20, 21, 22. We would call such PTM types canonical further below. Most prominently, the N‐ and the C‐termini of proteins are candidates for such PTM sites, due to their nature of being located at flexible protein extremities, often with the role of anchors to execute their targeting function properly 23.

The same consideration renders most of the globular regions essentially non‐eligible for enzyme‐generated PTMs. Nevertheless, there are several such cases known in true tertiary structural segments as partially reviewed in 10. They can be classified as (i) instances of autocatalysis/self‐processing (the modified protein is also the modifying enzyme), (ii) PTMs in large loops and/or of very exposed residues with long side chains, (iii) instances executed before/during protein folding or during periods of temporary unfolding or (iv) spontaneous PTMs caused by scavenging action of small reactants that can diffuse into the 3D structure of the substrate protein, often as a result of their elevated concentration or of long exposure time frames.

In this study, we report the results of our large‐scale analysis of single‐residue PTMs that have been systematically categorized by UniProtKB under their controlled vocabulary and the relationship of the sequence positions with regard to regions of known or predicted 3D structures. Thus, we systematically analyzed the likelihood of a PTM occurring in exposed, accessible segments of protein sequences. Special attention has been paid to PTMs that occur at the proteins’ N‐ and C‐termini. The goal is to work towards a catalogue of PTM types that can occur in tertiary structure regions that do not provide obvious access to a modifying enzyme (in contrast to the list of PTMs that are located in non‐globular sections of the sequence which are readily accessible to the modifying enzymes). To note, each observation of a chemically modified residue in non‐accessible areas of the protein structure requires answering the question about the underlying biology including its genesis mechanism. What are the non‐trivial biological circumstances that provided the opportunity for this PTM to come into existence?

## 2 Materials and methods

In this work, we used 540 958 protein entries from UniProtKB/Swiss‐Prot Release 2013_09 4 and extracted all sequences that carry single‐residue PTM information. Our cases of interest were all of those annotated in UniProtKB/Swiss‐Prot under their FT lines with the key name MOD_RES or LIPID, both related to posttranslational modifications of single residues that have been systematically categorized by UniProtKB in their controlled vocabulary of posttranslational modifications. To test the coverage of these sites by sequence segments with known 3D structures, we searched for significant BLAST+ 24 hits (*E*‐value < 0.001) in the protein structure database of non‐redundant sequences (pdbaanr from 24 November 2013) provided by the Dunbrack Lab 25. Instead of limiting ourselves to PDB structures of known entries, BLAST+ was used in order to enlarge coverage, ensuring that proteins that did not have their structure experimentally solved, but are statistically significantly related to proteins of known structures, are taken into consideration in our analysis.

Because the structural database is made from sequences from the PDB SEQRES records, a significant BLAST+ hit does not guarantee that the atomic coordinates for the modified residue we are interested in have been really resolved. For this purpose, it is necessary to check the “REMARK 465” annotations in the respective PDB file. To circumvent this problem, an additional alignment step between the UniProtKB entry and the sequence created to consider only the residues whose CA (C‐alpha) atomic coordinates have been resolved, was done for every significant hit. By comparing these two alignments, we were able to identify whether the residue of interest was not only mapped to an available PDB structure 26, but also whether it had its atomic coordinates, at least, partially resolved.

**Figure 2:**
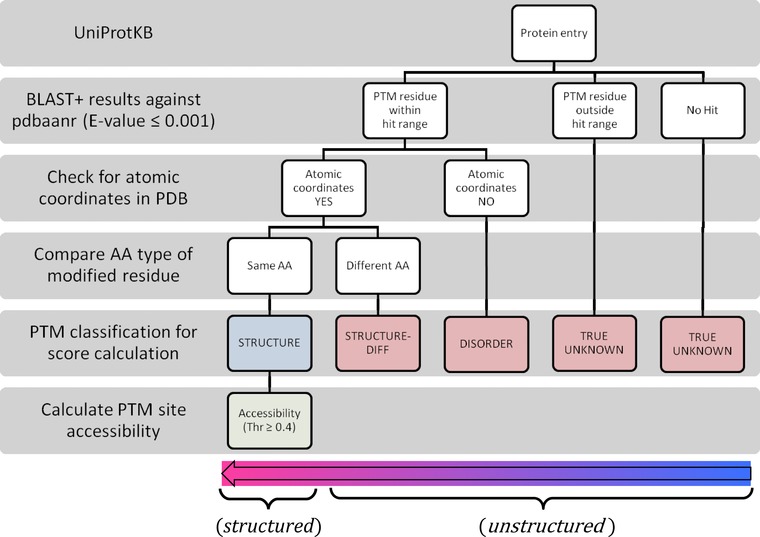
Protocol to classify UniProtKB PTM sites according to structure availability in the atomic coordinate level and their related score.

Following the mapping of every MOD_RES or LIPID PTM to a protein structure, we classified each one as (*a*) *STRUCTURE*, (*b*) *STRUCTURE‐DIFF*, (*c*) *DISORDER* or (*d*) *TRUE UNKNOWN* in this order of preference (see Fig. 2). If the residue of interest was mapped to an exact match against the PDB structure, i.e. the same amino acid type, we labeled it as *STRUCTURE*. If it found a hit (but to a different amino acid type), we called it *STRUCTURE‐DIFF*. The classification of *DISORDER* was given to cases where no atomic coordinate was available, despite of a significant hit to the PDB sequence (cases of sequence segments covered by REMARK 465 annotations), while *TRUE UNKNOWN* covered the cases where the query sequence did not find any significant hit. For every PTM we counted the number of classifications *N*_a_, *N*_b_, *N*_c_ and *N*_d_ above and calculated a score that ranges from –1 to 1 to estimate how many of the UniProtKB annotated PTMs are significantly associated to structures according to the equation below, where *N*_a_ represents the PTM *structural* number and (*N*_b_+*N*_c_+*N*_d_) the *unstructural* one. For the scoring purpose only, every classification that was not directly associated to a known structure with the specific amino acid type of the considered PTM, we referred to the *unstructured* contribution to the score.

(1) scor e PTM =Na−Nb+Nc+NdNa+Nb+Nc+Nd

Each classification count *N*_index_ was additionally split into N=N order +N disorder , to further analyze for disorder prediction.

As a positive score would only imply that a PTM was more often found to be associated with a known structure rather than provide information on how accessible the modified residue would be to its enzyme, the next step was to calculate the residue surface accessibility for the PTM residues with exact matches in the PDB. The surface area of a residue in the PDB 26 was calculated by using DSSP 27. In order to estimate its accessibility, the ratio between its surface area and that of its reference value was calculated. Here, we used the reference values calculated for every residue X in the tripeptide Gly‐X‐Gly according to Chothia 28; thus, actual accessibilities that are expected to be within the interval 0 and 1 might, in some cases slightly exceed 1 due to numerical issues (most predominantly for terminal residues since the main chain contributes more to the accessibility in these cases compared with the standard Gly‐X‐Gly situation). For every PTM, the average accessibility and its standard deviation were calculated.

In parallel, we attempted to quantify the amount of disorder in the sequence environment of PTM sites with two approaches. Disorder prediction was estimated by IUPred 29 using previously benchmarked parameters 30. An additional analysis to include disorder information from DisProt release 6.02, currently with 694 protein entries 31, was carried out. We tested whether the sequence segment from the DisProt contained any of the PTM sites (using the UniProtKB IDs supplied by DisProt) including its identical sequence environment.

## 3 Results

### 3.1 Generation of an electronically readable dataset of PTM sites in protein sequences and their coverage by known or predicted 3D structures

We have developed a computer‐supported workflow that allows systematic identification of posttranslational modification sites in UniProtKB 4 sequences, scoring them according to their coverage with 3D structures from PDB 26 and, if the site is part of a sequence segment corresponding to an available 3D structure, determining the average accessibility of the PTM instances in these structures. Since the requirement of absolute sequence identity between sequences in UniProtKB and PDB would exclude many hits and since considering structures known for homologous sequences open the opportunity to improve the structural coverage, we used a BLAST criterion for UniProtKB sequence – PDB structure alignment. To note, we take care (i) for differences in the residue type at the PTM site and in the sequence for which the 3D structure is known and (ii) for sequence ranges for which atomic coordinates are missing. When a significant structure was associated with a PTM site, average relative accessibility of the residue annotated to be modified was determined (as a score expected between 0 and 1 where a residue with relative accessibility ≥0.4 is considered not buried). Details are described in Section 2 and Fig. 2.

In this work, we apply a 3D structure score (Eq. (1)) for measuring how often we find a given PTM type associated with a sequence segment covered by a known protein structure in the PDB. This score ranges from –1 to 1, meaning that a score of –1 indicates that the protein sequence where the PTM annotation is found shows no sequence similarity to available protein structures in the PDB, while a score of 1 indicates that all sequences with the annotated PTM found a significant hit to at least one protein structure. Zero scores represent that half of the PTM annotated proteins found at least one significant structural hit, while the other half found none. It should be emphasized that our method to compute the 3D structure coverage leads rather to an underestimation of the 3D structure score and, therefore, the list of PTM sites buried into 3D structures is most likely not complete.

Once all the computations had been completed, we searched for PTM types with a high 3D structure score and low accessibility of the residue to be modified. At this stage, manual interference is required to clarify the biological plausibility of the findings with analysis of the respective 3D structures and the scientific literature about the sequence targets. Therefore, we had to restrict the volume in this work and concentrated on PTMs described with key names “LIPID” and “MOD_RES” only; thus, all PTMs with carbohydrates were omitted from consideration. To note, carbohydrate‐type PTMs constitute 38% of all PTM annotations supplied by UniProtKB (the totality of all LIPID, MOD_RES and carbohydrate annotations). The data files generated for all PTM types studied can be obtained on request from the authors. For the sake of presentation, it is necessary to classify the PTM types with regard to their localization in the protein sequence (at the N‐terminus, at the C‐terminus and somewhere in‐between). We followed the definitions from UniProtKB 4 as provided in the file “ptmlist.txt” as from 18th September 2013.

### 3.2 Lipid PTMs: overview

**Figure 3:**
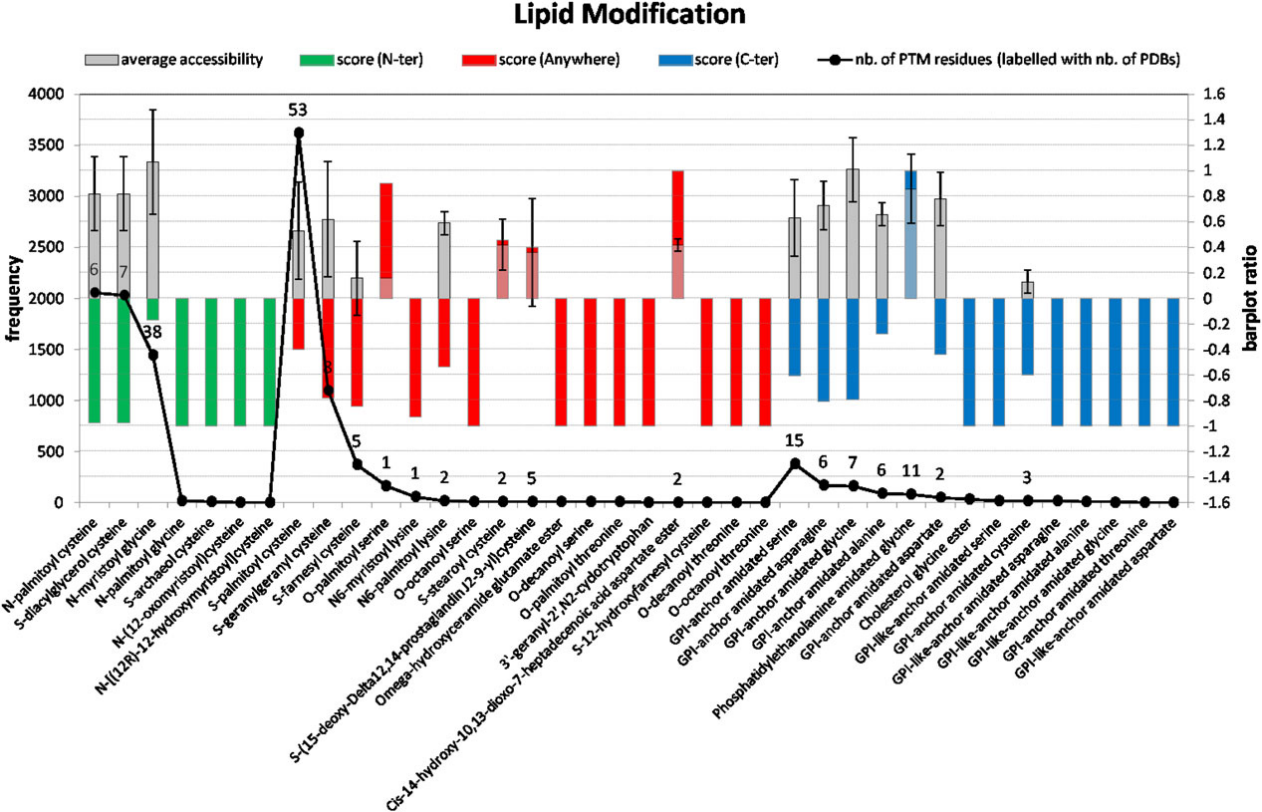
Lipid modifications analysis. The three colored bar plots (green, red and blue to define the PTM location for N‐terminal, anywhere and C‐terminal, respectively) display the calculated score that ranges from –1 to 1. A score of 1 represents that all proteins with the given PTM are associated to at least one known structure through BLAST search, as described in the methodology section. The curve depicting the number of modifications in the database (left *y* axis) is labeled with the number of PDBs found. The gray bar plot represents the average accessibility with its standard deviation of the modified residue calculated from the identified PDB structures, as described in methodology.

In Fig. 3, we present the absolute coverage of sites for lipid PTM types with sequence segments from known 3D structures, the respective 3D structure scores as well as the relative accessibility (mean and standard deviation). Overwhelmingly, we see large negative 3D structure score (green, red and blue bars into negative scores, Fig. 3). If there is structure coverage (mostly, without the actual lipid modification), it is typically accompanied with high accessibility of the residue to be modified. Thus, we can assume that lipid PTMs, as a rule, occur in regions without preferred 3D structure and the conditions for enzymatic PTM are fulfilled. There are a couple of exceptions that require further analysis. As will be visible below, we can provide plausible arguments in essentially all cases that argue for a residue to be lipid modified in a state that is accessible to an external enzyme.

It should also be emphasized that the number of annotated lipid modifications for residues far away from either termini with positive scores is typically very small in the UniProtKB. The only notable exception is O‐palmitoyl serine PTM that is featured in 164 annotations. This is another indication that lipid modifications preferentially occur at the generally more accessible protein N‐ or C‐termini 23, either at the outright terminal position or at some residue nearby. In the case of acylation, the role of the modifying enzyme might be reduced to just some mildly facilitating role compared with other cases of lipidation. It is known that acyl‐coA has quite some reactivity on its own that can lead to spontaneous acylation in *in vitro* conditions 32, 33. This might be a reason why some limited accessibility of the cysteine in question might suffice in these cases and certain acyltransferases rather have the role of positioning the acyl‐coA appropriately near the receiving residue 34, 35, 36.

### 3.3 Lipid PTMs: N‐terminal cases

While we observe hardly any 3D structure coverage for some N‐terminal acylation sites (score close to –1 for N‐palmitoyl‐cysteine and S‐diacylglycerol‐cysteine), others have more structure hits such as the N‐myristoyl‐glycine PTM (score –0.17) but accessibility of the residue to be modified is high in all cases. It should be noted that, in the case of N‐terminal myristoylation, the complete sequence motif consist of a 6‐residue N‐terminal stretch that enters the N‐myristoyl‐transferase active site and an adjacent ∼11 residue linker regions 14, 37. Not surprisingly, this sequence segment is missing in a number of 3D structures of myristoylated proteins partially or completely.

### 3.4 Lipid PTMs: C‐terminal cases

In the case of phosphatidylethanolamine‐amidated glycines, all the proteins, totalizing 81 annotations for this PTM, found a significant BLAST hit to at least one of 11 PDB structures according to our method. All the identified structures displayed a highly accessible C‐terminal (accessibility >0.8), in agreement with the idea of accessibility being a pre‐requisite for PTMs catalyzed by enzymes (see also Supporting Information Fig. 1).

For several otherwise GPI lipid anchored proteins, 3D structures (naturally, without the anchor itself) are available that also cover the ω‐site, the site of anchor attachment 38. In the case of GPI lipid anchored amidated glycine, the 3D structure score is even positive and close to 0.9. Yet, the accessibility of the ω‐site residue is high in all cases except for GPI lipid anchored amidated cysteine. In the latter instance, the respective sequences found significant BLAST hits to three structures. Two of them matched the transferrin‐like 2 motif of lactoferrin (1B1X:A, 39) and human transferrin (3VE1:B, 40). Despite this similarity to other transferrins, only melanotransferrin is annotated to be a GPI‐anchor amidated cysteine protein, as it has been shown to be expressed at the cell surface with this modification 41. As recently reviewed 42, the role of melanotransferrin is still elusive despite its implication in malignant melanoma.

The coverage with 3D structures in these cases does not constitute a mechanistic paradox. It should be noted that, in vivo, GPI lipid anchored proteins are translated together with a C‐terminal propeptide that is cleaved by a transamidase in the endoplasmic reticulum 38, 43, 44. The C‐terminal sequence motif includes a ∼10 residue linker‐type region, a 4‐residue cleavage region (residues –3…+1 with the ω‐site at position 0) and a ∼6 residue spacer region followed by ∼20 residue hydrophobic segment 12, 38. This PTM site motif architecture provides the required accessibility for the enzymatic transformation.

### 3.5 Lipid PTMs: any, in general non‐terminal sequence position

The majority of lipid modifications occurring anywhere had a negative 3D structure score (13 out of 17 red bars in Fig. 3). Among these, eight did not find any hit to a 3D structure. In three further cases (S‐palmitoyl cysteine, N6‐palmitoyl lysine and S‐geranylgeranyl cysteine), the accessibility of the residue to be modified is far above the threshold 0.4. Two cases (S‐farnesyl cysteine, N6‐myristoyl lysine) have a negative 3D structure score with low accessibility of the PTM residue in the single or the few structures found. Four further lipid PTMs (cis‐14‐hydroxy‐10,13‐dioxo‐7‐heptadecenoic acid aspartate ester, O‐palmitoyl serine, S‐stearoyl cysteine and S‐(15‐deoxy‐Delta12,14‐prostaglandin J2‐9‐yl)cysteine) have a positive 3D structure score and a residue accessibility below or close to the threshold used (0.4). Comments for these six cases are below.

#### 3.5.1 S‐farnesyl cysteine PTM

Among the five protein structures found, four belong to the Ras superfamily. Many superfamily members are known to undergo in vivo verified C‐terminal prenylation (farnesylation or geranylgeranylation) 45, 46. To note, the general prenylation sequence motif consist of a small 3–4 residue cysteine‐containing stretch at the ultimate C‐terminus with an adjacent segment of ∼10 residues having linker‐type properties 15. As our sequence similarity search found significant hits to Ras‐type structures, it was important to understand why low accessibility was observed for these few cases (see Supporting Information Fig. 2). Structural overlap of the examples with buried cysteines revealed that, the otherwise modified cysteine (Cys188 in the respective PDB structures, e.g. 2NGR:A 47), forms an intramolecular disulfide bond with Cys105. This form has been proposed to represent an alternative mechanism for retaining a cytosolic pool of the G protein, but is unlikely to be present in vivo 47. The fifth structure (4DM9:A, 48, see Supporting Information Fig. 3) was found with the potential modification site (without lipid anchor) not easily accessible; yet, there is experimental evidence for farnesylation 49. It appears that this situation implies the requirement of partial unfolding and/or some structural differences under in vivo conditions.

#### 3.5.2 N6‐myristoyl lysine PTM

We found a generally negative 3D structure score. The only structural hit indicates low accessibility. Cytochrome c oxidase subunit 1 (COX1) has been reported to atypically incorporate myristic acid at an internal lysine found within one of the predicted transmembrane helices of subunit 1 50, justifying its low accessibility in the PDB structure available (Lys319 in 3AG3:A 51). There are only a few cases of N6‐myristoylation of lysines reported 14 and the biological mechanism involved remains enigmatic.

#### 3.5.3 A S‐(15‐deoxy‐Delta12,14‐prostaglandin J2‐9‐yl)cysteine PTM

Among all the PTMs studied, this one has the largest standard deviation in relative accessibility. The structural hits are divided into 2 distinct groups of proteins structures: the epoxide hydrolases (3I28:A 52, 1CR6:A 53, 4JNC:A 54) having cysteines with low accessibility on the one hand and the p50 subunit of NF‐kappa B transcription factor (2O61:B 55) and the transcription repressor Rex (2VT2:B 56) with high accessibility on the other. However, redox regulation of soluble epoxide hydrolase by 15‐deoxy‐delta‐prostaglandin J2 has been shown to be important in the control of physiological response of coronary hypoxic vasodilation 57. Since all the three epoxide hydrolase structures do not contain the lipid modification, it would be plausible to expect the segment carrying the cysteine to be more tightly packed against the core within the crystal than it might be in vivo during the modification process. GTPase H‐Ras proteins from human, mouse and rat have also been annotated to receive this lipid modification. These entries, however, did not find any structural evidence for the cysteine site. In fact, the human protein has been assigned to a disorder region in DisProt 31.

#### 3.5.4 O‐palmitoyl serine PTM

All proteins annotated belong to the signal transducing Wnt family. The majority (156 out of 164) found a significant hit to a single 3D structure, that of *Xenopus laevis* Wnt8 in complex with the cysteine‐rich domain of Frizzled 8 (4F0A:B 58). Thus, this explains the high positive 3D structure score observed for this PTM. However, low cysteine accessibility is an artifact in this case since Wnt8 is in a complex with Frizzled8 in the structure. The modified Ser187 is located in a protruded loop, also known as the tip of the Wnt's thumb, where the palmitoleic acid lipid is projected into a deep groove in the Fz8‐CRD (see Supporting Information Fig. 4).

**Figure 4:**
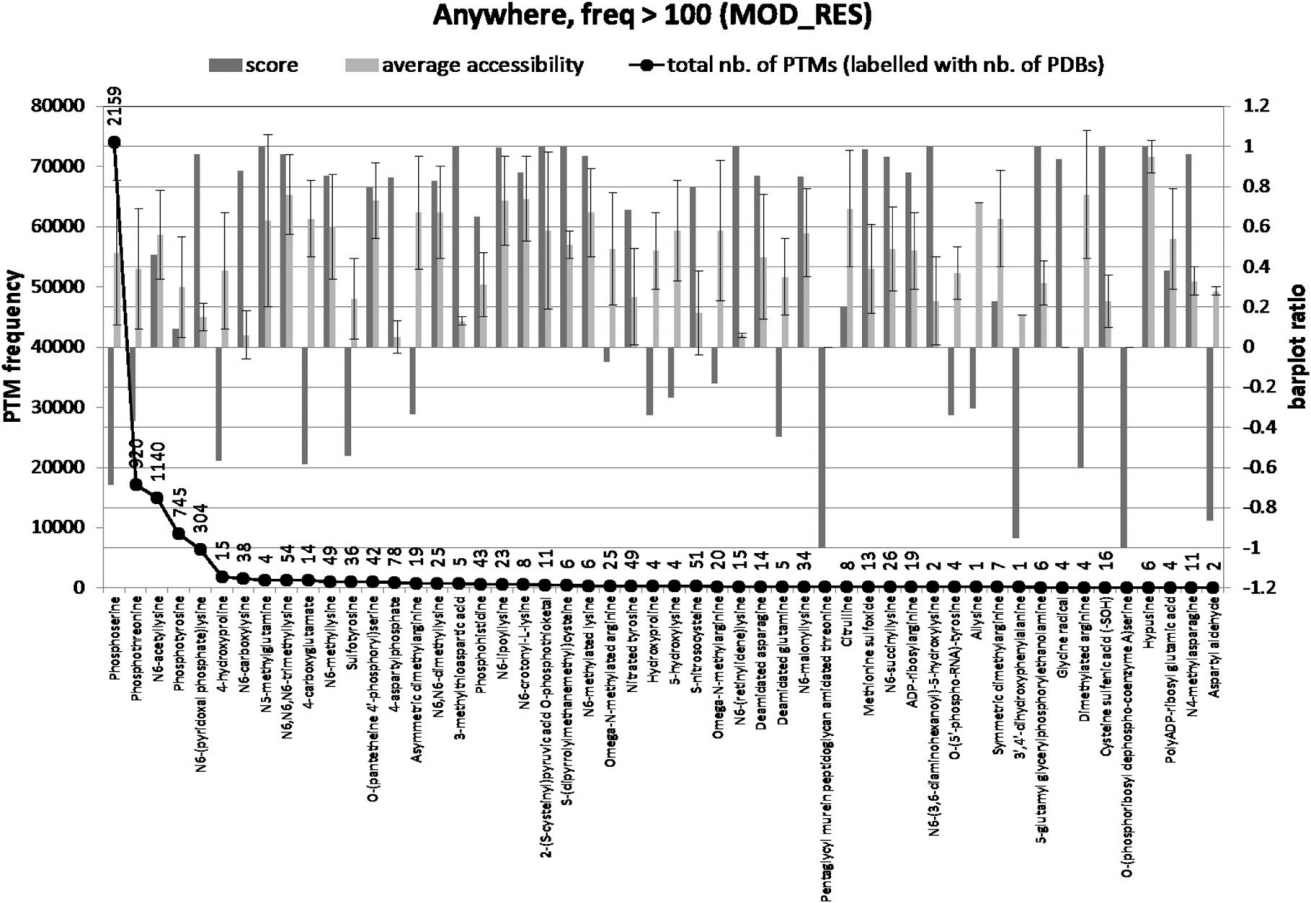
Most frequently annotated PTMs of MOD_RES occurring anywhere. Threshold for frequency annotation was >100. The bars in darker gray display the calculated score that ranges from –1 to 1. A score of 1 represents that all proteins with the given PTM are associated with at least one known structure through BLAST search, as described in the methodology section. The curve depicting the number of annotated modifications in the database (left *y* axis) is labeled with the number of PDBs found. The light gray bar plot represents the average accessibility with its standard deviation of the modified residue calculated from the identified PDB structures, as described in methodology.

#### 3.5.5 S‐stearoyl cysteine PTM

8 out of 11 annotated proteins were hit by two distinct PDB structures. Among these eight sequences, seven are viral proteins and are similar to the 3D structure of 3J2W:E 59. Structural evidence indicates that the modified residue is at the cytosolic end of a transmembrane helix (see Supporting Information Fig. 5). However, it should be noted that the residue for the S‐stearoyl cysteine lipid modification has been described as palmitoylated elsewhere 60. The other sequence, human P‐selectin, found a significant hit to the crystal structure of human apolipoprotein‐H (1C1Z, 61). Different structural regions of human P‐selectin have been elucidated, but not the region where the PTM lies within. The matched cysteine in the structure found makes a disulfide bond with the terminal cysteine (see Supporting Information Fig. 6).

**Figure 5:**
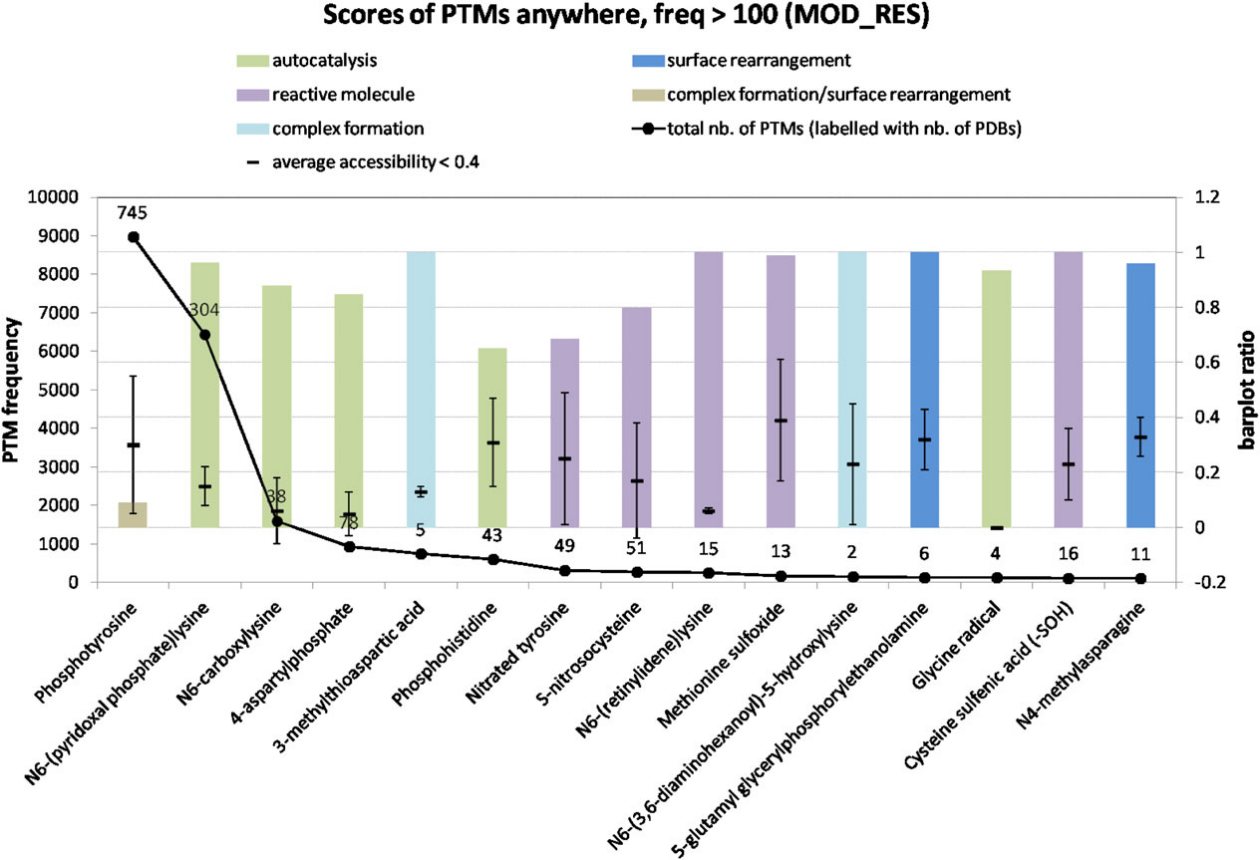
Most frequently annotated PTMs of MOD_RES occurring anywhere in the sequence with a positive score and low accessibility for the PTM residue. Threshold for frequency of annotation was >100. The colored bar plot displays the positive scores up to 1, according to the classification of Table 2. A score of 1 represents that all proteins with the given PTM are associated to at least one known structure through BLAST search, as described in the methodology section. The curve depicting the number of annotated modifications in the database (left *y* axis) is labelled with the number of PDBs found. The plot with the symbol (**–**) represents the average accessibility with its standard deviation of the modified residue calculated from the identified PDB structures, as described in Section 2. Only the cases where the average accessibility value was below 0.4 are represented in this figure.

#### 3.5.6 Cis‐14‐hydroxy‐10,13‐dioxo‐7‐heptadecenoic acid aspartate PTM

This ester was only annotated for two proteins, both known as non‐specific lipid‐transfer proteins found in wheat and barley. The annotated aspartate is exposed and a probable moiety for binding lipids 62 (see Supporting Information Fig. 7).

### 3.6 MOD_RES annotated PTMs: Overview

Among all 275 MOD_RES PTMs in the controlled vocabulary of UniProtKB released in September 2013, 24 of them did not have their position of the modification in the polypeptide field (PP) described, 41 were described as N‐terminal, 35 of them as C‐terminal, 148 as anywhere in the sequence and 27 as “protein core”. First, we analyze the 76 terminal and the 148 anywhere occurring PTM types. The 24 cases without positional characterization and the 27 “protein core” cases will receive attention in the last part of this section.

Table 1A (for terminal PTMs) and 1B (for all PTM types described as anywhere in the sequence) provide details of the breakdown of the first three PTM types with regard to 3D structure score and average relative accessibility. This process facilitates the identification of exceptional cases for further investigation. Overwhelmingly, the 3D structure score is negative and the accessibility is high for N‐ and C‐terminal PTMs (70%). For another 14%, the sequence stretch is generally covered by a 3D structure but the residue to be modified is widely accessible. Thus, 84% of the terminal PTMs is clearly of the canonical type. For the other PTM types, this can be said for at least 58% of them.

**Table 1:** Frequency of PTMs annotated under MOD_RES taking into account (A) terminal residues only and (B) anywhere

		Accessibility		
		<0.4	≥0.4 or n.a.	Total
*A*				
Score	Positive	8 (11%)	11 (14%)	19 (25%)
	Negative	3 (4%)	53 (70%)	56 (74%)
	Zero	1 (1%)	0	1 (1%)
	Total	12 (16%)	64 (84%)	76 (100%)
*B*				
Score	Positive	54 (36.5%)	36 (24.3%)	90 (60.8%)
	Negative	7 (4.7%)	50 (33.8%)	57 (38.5%)
	Zero	1 (0.7%)	0	1 (0.7%)
	Total	62 (41.9%)	86 (58.1%)	148 (100%)

In Fig. 4, we present the absolute coverage of sites for most frequently occurring non‐terminal MOD_RES annotated PTM types with similarity to at least one sequence segments from known 3D structures, the respective 3D structure scores as well as the relative accessibility (mean and standard deviation). Overwhelmingly, we see that, even in cases of positive 3D structure score (dark grey bars, Fig. 4), the accessibility is high. Thus, we can assume that also in this class of PTMs, as a rule, the sites occur in accessible regions and the conditions for enzymatic PTM are fulfilled.

### 3.7 MOD_RES annotated PTMs: N‐ and C‐terminal PTMs with low relative residue accessibility

This minority of PTM types can be further analyzed in terms of a positive (11%), negative (4%) or zero (1%) 3D structure score. Negative scores indicate that 3D structural evidence covering the PTM site is not available for most of the annotated entries and there might be a single PDB structure for a couple of sequences.

This situation occurred for three PTMs: N‐methylleucine (3QT4:A 63), N‐methylproline (4JHP:C 64) and N‐acetylproline (3KER:A, 3KAN:A; Zierow et al., to be published) and are summarized in Table 1 of Supporting Information. Further investigation of the few mapped structures for these PTMs revealed that not only a single annotated PTM from each *N‐methylleucine* and *N‐methylproline* was sufficient to be mapped to a single structure each: 3QT4:A and 4JHP:C, respectively, but that the mapping of their modified N‐terminal residue was done to a non‐terminal one, either because modification occurs after protein maturation or because mapping was done to a more repetitive fold such as the RCC1‐like, allowing multiple BLAST region hits.

Notably, N‐terminal acetylation is a PTM that can increasingly routinely be handled with available experimental protocols 65. One specific form, *N‐acetylproline*, was the other interesting case given that only 8 out of 315 annotations were mapped to the two PDB structures 3KER:A and 3KAN:A. These structures, despite having a proline at its N‐terminal, were elucidated with a chemically blocked N‐terminal. Consequently, cases with few structural outliers like this one can be easily interpreted as artifacts due to the experimental structure determination with unnatural modifications.

*Pyruvic acid* (reaction starting for serine) was the only case with zero score (see Supporting Information Table 2), meaning that half of these annotated PTMs (611) found a significant hit to at least one PDB structure. These could easily be clustered into three different structural groups, all belonging to decarboxylases. The first group encompasses five of these PDB structures and they belong to the Aspartate decarboxylase protein family (1PQF and 3OUG 66, 1PPY 61, 4AZD 67, 2C45 68). These have been demonstrated to involve a self‐processing reaction. The second group has four structures belonging to the adenosylmethionine decarboxylase family (1JL0 69, 2III (Kanaujia et al., to be published), 1TLU 70 and 1VR7 (to be published)) and are also involved in autocatalytic processing of a precursor protein 71. The last group is represented by a single structure belonging to the pyruvoyl‐dependent arginine decarboxylase family (2QQD:C 71, 72). Again, the PTM pyruvic acid (from serine) has been verified to result from self‐processing reactions.

**Table 2:** Classification based on literature and structural analysis of most frequent MOD_RES PTMs with positive scores under UniProtKB modification field “anywhere” and “protein core” with regard to their mechanism of genesis

PTM	Classification	No. of	Score	Localization	Accessibility	Nb. of
		instances				PDB hits
Phosphotyrosine	Complex formation/surface rearrangement	8970	0.09	Anywhere	0.3 ± 0.25	745
N6‐(pyridoxal phosphate)lysine	Autocatalysis	6443	0.96	Anywhere	0.15 ± 0.07	304
N6‐carboxylysine	Autocatalysis	1600	0.88	Anywhere	0.06 ± 0.12	38
4‐aspartylphosphate	Autocatalysis	927	0.85	Anywhere	0.05 ± 0.08	78
3‐methylthioaspartic acid	Complex formation	743	1.00	Anywhere	0.13 ± 0.02	5
Phosphohistidine	Autocatalysis	601	0.65	Anywhere	0.31 ± 0.16	43
Nitrated tyrosine	Reactive small molecule	325	0.69	Anywhere	0.25 ± 0.24	49
S‐nitrosocysteine	Reactive small molecule	267	0.80	Anywhere	0.17 ± 0.21	51
N6‐(retinylidene)lysine	Reactive small molecule	245	1.00	Anywhere	0.06 ± 0.01	15
Methionine sulfoxide	Reactive small molecule	175	0.99	Anywhere	0.39 ± 0.22	13
N6‐(3,6‐diaminohexanoyl)‐5‐hydroxylysine	Complex formation	152	1.00	Anywhere	0.23 ± 0.22	2
5‐glutamyl glycerylphosphorylethanolamine	Surface rearrangement	124	1.00	Anywhere	0.32 ± 0.11	6
Glycine radical	Autocatalysis	123	0.93	Anywhere	0	4
Cysteine sulfenic acid (‐SOH)	Reactive small molecule	112	1.00	Anywhere	0.23 ± 0.13	16
N4‐methylasparagine	Surface rearrangement	104	0.96	Anywhere	0.33 ± 0.07	11
2,3‐didehydroalanine (Ser)	Autocatalysis	292	0.75	Protein core	0.19 ± 0.05	3
1‐thioglycine	Complex formation	86	0.91	Protein core	0.88 ± 0.64*	9
2,3‐didehydroalanine (Cys)	Complex formation	35	1.00	Protein core	0.66*	1

“Surface rearrangement” implies unfolding to make the site accessible to an external enzyme. For PTMs under “anywhere”, only the low accessibility cases, as in Fig. 5, were considered. Asterisk (*) represents values that were above the accessibility threshold to be considered as buried.

*N,N‐dimethylleucine* PTM has only one annotated case, matching the previously described N‐methylleucine for the same residue in the same protein (Supporting Information Table 2).

*5‐glutamyl 2 aminoapidic acid, 5‐glutamyl N2‐lysine (or –arginine or –glutamate)* PTMs can be structurally related to the residue 56 in 3VPB:E 73, 74, 75, the alpha‐aminoadipate/glutamate carrier protein LysW (Supporting Information Fig. 8).

*CysO‐cysteine adduct* is annotated for only two mycobacterium proteins that participate in the cysteine biosynthesis. The modified C‐termini are clearly hidden in the protein complex involved in amino acid biosynthesis for both cases 73, 74, 75 (see Supporting Information Fig. 8) and are widely accessible if the protein is kept outside the complex.

*Leucine methyl ester* and *Glycyladenylate* PTM cases repeat the low accessibility artifact due to complex formation after residue modification (Supporting Information Fig. 8). Basically, these are the only two terminal PTMs that were frequently annotated in UniProtKB and having low relative accessibility and a positive 3D structure score. Both of them had their modified terminal inserted into one of the complex subunits. Structural mapping of Leucine methyl ester yields three PDB structures (3FGA:C, 3C5W:C and 3P71:C) representing complexes with PP2A (Protein phosphatase 2A catalytic subunit) 76, 77, 78. Leucine methyl ester formation requires a PP2A‐specific methyltransferase enzyme. Two of these structures were solved in the presence of the enzyme 76, 77, while the third was solved in the complex PP2A‐shugoshin 78. Glycyladenylate annotations have been matched to the sulfur carrier subunit of a few different proteins, where C‐terminal thiocarboxylation is a necessary step for the active site formation 79, 80.

In summary, with the exception of the PTM pyruvic acid (from serine) that has evidence for participating in a self‐processing reaction, all other PTMs annotated to terminal residues were highly exposed, at least if interaction partners that form complexes after the PTM formation have been removed.

### 3.8 MOD_RES annotated PTMs: non‐terminal PTMs with low relative residue accessibility but negative 3D scores

There were very few cases of negative 3D scores with low accessibility that are further addressed below.

#### 3.8.1 3’,4’‐dihydroxyphenylalanine PTM

Despite UniProtKB having 130 proteins annotated with this modification, only 3 UniProtKB entries were structurally mapped to one single PDB: 1NGK 81, the Hemoglobin‐like protein HbO. The modified residue in this structure is not accessible, as it plays an important role in the ligand binding and on the heme distal site architecture 82.

#### 3.8.2 Sulfotyrosine PTM

Despite being a very common modification for secreted or membrane‐bound proteins 83, the human tyrosylprotein sulfotransferase‐2, one of its catalyzing enzymes, has only been recently elucidated 84. We have found 36 PDBs for entries with this modification, ranging from peptides (conotoxins) to proteins belonging to different Pfam families such as transmembrane receptor from rhodopsin family, carboxylesterase family, fibronectin domains, Leucine rich repeats, multicopperoxidade, peptidases, among others.

#### 3.8.3 Aspartyl aldehyde PTM

Only seven protein entries were associated to two structures (3ARC:C 85,3A0B:C 86), both of them belonging to Photosystem II complex. The Photosystem II CP47 reaction center protein of Spinach (accession: P04160) has been shown to undergo this modification 87. However, only proteins belonging to other plants and algae showed similarity evidence to the structure of this reaction centre from *Thermosynechococcus vulcanus*.

#### 3.8.4 Deamidated glutamine PTM

56 proteins entries with this modification were mapped to 5 PDBs. It is interesting to note that one of this, somatotropin, a human growth hormone (accession P01241), linked to the structure 1HWG:A 88, both in our findings and in UniProtKB, has been verified to undergo this modification 89. For the purpose of accessibility calculation, however, its glutamine side chain has been resolved for backbone and C_β_ atoms only, consequently producing a fictitious lower accessibility number in this example. Occurrence of this PTM in disordered regions has also been previously mentioned 90.

#### 3.8.5 O‐(5’‐phospho‐RNA)‐tyrosine

50 protein entries, all belonging to genome polyproteins from Caliciviridae and Enterovirus, found one of the four structures of viral protein genome‐linked (VPg), which is inherent to this modification. Three of them were resolved by NMR 91, 92, while one was crystallized in the presence of the RNA‐dependent RNA polymerase 93, demonstrating the usage of different binding sites for VPg uridylylation, which is the process where the hydroxyl group of the tyrosine residue from VPg is covalently linked to two UMP molecules by the RNA‐dependent RNA polymerase.

#### 3.8.6 4‐hydroxyproline PTM

This is a very common modification observed in eukaryotes ranked at the eighth most annotated PTM MOD_RES in our study with a total of 1806 annotations. It is interesting to highlight the PDB 3HQR 94 among the only 15 PDBs found, where the PHD2 catalytic domain (Prolyl Hydroxylase Domain) and its substrate peptide, the C‐terminal degradation domain (CODD) of Hypoxia‐inducible factor 1 alpha, have been resolved. This structure elucidation played an important role in the understanding of the structural basis of peptide hydroxylation by the PHD family 95. Its average accessibility in our study (0.38) was very close to the threshold used (0.4) presenting a considerable standard deviation (±0.29) due to the different nature of the structures found.

#### 3.8.7 Phosphothreonine PTM

It is the second most annotated MOD_RES PTM in the database with 17 172 annotations associated to 920 PDB structures. Similarly to 4‐hydroxyproline described above, its average accessibility among all these structures was very close to the threshold (0.39), also with a considerable standard deviation (±0.3).

It appears that the examples discussed in this section belong either to the group of PTMs catalyzed by an external enzyme or they involve autocatalysis/self‐processing. The aspartyl aldehyde PTM is most unclear but it might also be the result of an autocatalytic process.

### 3.9 MOD_RES annotated PTMs: non‐terminal PTMs with low relative residue accessibility but positive 3D scores

Interestingly, for positive scores, the number of PTMs mapped to structural sites with low accessibility was higher (54) than those mapped to more exposed ones (36). In order to better analyze this scenario, we first looked at how often a PTM occurred, in order to identify if these buried sites were among highly annotated PTMs. In general, 41% of all MOD_RES PTM types occur less than ten times in the whole UniProtKB. The percentage of a given PTM type occurring >10 and <100 times was 24%. Consequently, only 35% of these PTMs were annotated more than 100 times. Among these 52 most frequently annotated ones, 18 have negative and 34 have a positive 3D structure scores. Among the latter 34 cases, 19 have an average accessibility value above the threshold of 0.4, while this value is below 0.4 for the other 15. In Fig. 5, we list these 15 frequently annotated UniProtKB PTM types that are associated with buried sites in 3D structures mapped to the annotated protein sequences.

*Phosphohistidine* is an interesting example found in this group (Fig. 5). Bacteria, plants and fungi have well characterized examples of histidine phosphorylated, so‐called two‐component systems, while our knowledge of the role played by histidine in mammalian systems is much less advanced 96. In our work, two UniProtKB Pyruvate Dehydrogenase Kinases, annotated to have the modification of phosphohistidine by autocatalysis (accessions Q9SBJ1 and Q9P6P9, from plant and yeast, respectively), found the structure of PDK1_HUMAN (accession Q15118). Currently, there is no information of a potential role for the histidine in this human isoform that could be phosphorylated. However, with recent advances for direct detection of protein histidine phosphorylation 97, there is good perspective for the elucidation of this role and our work does point out interesting cases for further verification.

Both *phosphohistidine* and *phosphotyrosine* are examples of phosphorylation with positive 3D score (Fig. 5). The work of Gao and Xu described that phosho‐serine/‐threonine/‐tryrosine have preference for occurring in disordered regions 90. Our data agrees with this information for phosphoserine and phosphothreonine only, as we identified more significant 3D structural coverage for phosphotyrosine, although one could argue that its score was close to zero (0.1), which describes a half/half scenario. Phopho‐Ser/Thr‐binding domains, such as WW domains, forkhead‐associated (FHA) domains, WD40 repeats, Polo‐box domains (PBDs), among others, are crucial in controlling cycle progression and DNA damage signaling 98. Phosphotyrosine binding domains, such as SRC‐homology 2 and PTB domains, have long been described to mediate protein‐protein interaction 99. Many of the phosphorylation instances are reported in sequences containing kinase domains representing either cases of autophosphorylation or modification by other kinases. Currently, the need to integrate the increasing knowledge on the phosphorylation machinery and its interconnections imposes a real challenge 100. Given the differing repertoire of binding domains, it is not surprising to see different accessibility numbers for the different phosphorylation types and also the study bias of different kinases may have contributed to different availability of associated structures (Supporting Information Fig. 9). The recent work of Vandermarliere and Martens pointed out to the necessity of analyzing phosphorylation sites in available structures for better account, as some buried sites could easily become exposed upon small conformational changes, while others would require an unfolded state, questioning the veracity of the site 101.

60% of the structures found in association with *N6‐(pyridoxal phosphate)lysine PTM* were resolved in the presence of PLP ligand (pyridoxal‐5’‐phosphate). Analysis of some of its structures revealed the non‐accessibility of the modified lysine, which serves as a cofactor for various reactions 102, 103, 104, 105.

*N6‐carboxylysine PTMs* are often found as part of or near the active site of enzymes, such as alanine racemase 106, OXA‐1 beta‐lactamase 107 and pyruvate carboxylase 108.

*4‐aspartylphosphate* has preference to occur in ordered regions according to Gao and Xu 90 and in the protein core according to Pang et al. 109, in agreement with our results. Bacteria use aspartyl phosphate for signalling as part of a two‐component regulating system 110, 111.

*3‐methylthioaspartic* acid is a modification that characteristically occurs on a conserved aspartate in ribosomal protein S12 in several bacteria 112. Structurally, the residue is buried in the ribosomal RNA‐protein complex at the internal binding interface facing the RNA. Enzymes important for this modification have been discovered 112, 113, 114, but they can logically only access the residue if the protein would be in its RNA‐unbound form.

Oxidative stress results in reactive nitrogen species which mediate *tyrosine nitration* that is a marker of several different diseases 115. The nitration reaction is not known to be enzymatic 116 and the highly reactive small peroxynitrite radical molecule may reach tyrosines also within protein structures without the need of an enzymatic access, which explains the positive score in our study.

Current data suggest that different mechanisms exists for the formation (and removal) of *S‐nitrosocysteine* in vivo, some requiring protein–protein interactions 117 and consequently accessibility of the modification site. Still, its biochemical mechanisms of formation, its effects on protein stability, the structural elements for its selection, as well as its molecular mechanisms where the PTM regulates protein function, remain uncertain 117. In agreement with Gao and Xu 90, our data supports its occurrence in structural regions of proteins that could aid in further investigation. For example, the structure of the human DNA repair endonuclease HAP1 118 shows the S‐nitrosocysteine sites of cysteine 65 (alternate), 93 (alternate) and 310 not accessible to solvent. S‐nitrosation modification of this protein in Cys93 and Cys310 was shown to play an important role in nuclear‐cytoplasmic translocation 119.

Proteins with the *N6‐(retinylidene)lysine PTM* play a very important role in the vision process throughout the animal kingdom 120. All the 15 structures found belong to the CATH 121 superfamily Rhopdopsin 7‐helix transmembrane proteins. This looks like an example of a non‐enzymatic reaction lying in the core of a transmembrane protein.

*Methionine sulfoxide* is a PTM associated to aging and pathophysiological conditions, as a consequence of methionine oxidation 122, a common spontaneous mechanism, unavoidable under aerobic conditions. The average accessibility that we have calculated is very close to the threshold with a considerable standard deviation (0.39 ± 0.22), ranging from buried to exposed. The localization of methionine within structures seems to be important in determining its accessibility to oxidants, contributing to complex redox‐regulation mechanisms of protein function, as well as eventually preventing other functionally relevant amino acids from being oxidized 122. This helps in understanding the range in accessibility observed for this residue that is a potential target to this PTM. Once modified, methionine sulfoxide reductases are necessary for the reduction step.

*N6‐(3,6‐diaminohexanoyl)‐5‐hydroxylysine PTM* had 152 instances annotated. They were all mapped to residue 34 of two structures of elongation factor P: 3A5Z:B 123 and 3TRE:A (Cheung et al., to be published). The accessibility of residue 34 is 0.2 and 1 in 3A5Z:B and 3TRE:A, respectively, because the first was resolved in the presence of a its putative lysyl‐tRNAsynthetase. Because 141 instances were mapped to 3A5Z:B, while 11 to 3TRE:A, we observed a bias in our Fig. 5 towards being buried, given that calculation was done considering all PTM instances. This is a clear example of an enzymatic PTM that has been resolved with and without the catalyzing enzyme producing a structural data discrepancy, easily understood through structural visualization (see Supporting Information Fig. 10).

Eukaryotic elongation factor 1A, another protein involved in the elongation steps of translation, is known to be modified in 2 conserved glutamate residues of mammals and plants with *5‐glutamyl glycerylphosphorylethanolamine* in domains II and III, while this is observed for a single conserved site for *Trypanosoma brucei* in domain III 124. Structural observation indicates that these residues are just partially buried on the protein surface becoming easily accessible (measured accessibility values ranging from 0.32 to 0.41, with residue 279 in domain II of 1B23:P 125 being the most buried (0.18), due to the surrounding side chains of adjacent residues).

*Glycine radical PTM* is found as part of the catalytic site of GREs (glycine radical enzyme), which are activated by GRE‐AEs (glycyl radical activating enzymes) 126. In our study, we identified the structures of PFL (pyruvate formate‐lyase) and GRE (Glycine Radical Enzyme), all having the same structural fold, as they belong to the radical S‐adenosylmethionine (SAM) superfamily 126. Insights into the structural basis for glycyl radical formation by PFL‐AE has been proposed 127. It is not surprising, though, that we observe this PTM being fully buried, due to the chemical nature of a glycine radical and its localization in this highly reactive catalytic site.

*Cysteine sulfenic acid* (‐SOH) is another example of a PTM resulting from oxidative stress. It has multiple roles in regulating protein function 128. This likely non‐enzymatic reaction requires the presence of reactive oxygen species that are small and can reach the modification sites even if partially buried.

*N4‐methylasparagine* has been identified in phycobiliproteins 129 and the catalyzing enzyme is known 50. In the structure, the modified residue is pointing to the chromophore and mutation of this residue causes changes in the photoreactivity of the protein complex 130. Although the residue is partially buried, it nevertheless resides within a surface loop which could rearrange to allow access by the methylating enzyme (Supporting Information Fig. 11).

The PTMs in this section are very diverse and include cases of (i) catalysis by an external enzyme, (ii) autocatalysis, (iii) reactions with small diffusible compounds (see also Table 2). The cases of tyrosine nitration, S‐nitrosocysteine and N6‐(retinylidene)lysine PTMs might require further scientific scrutiny though they appear to result from interactions with reactive small molecules diffusing into the structure.

### 3.10 MOD_RES annotated PTMs with the label “protein core” and without positional annotation

From all 275 MOD_RES PTMs in the UniProtKB controlled vocabulary, 41 were described as N‐terminal, 35 of them as C‐terminal, 148 as anywhere in the sequence, 24 did not have their position of the modification in the polypeptide field (PP) described and 27 were annotated as protein core. The latter two groups of PTMs will be discussed below.

All 24 PTMs without a localization description were described as a specific amino acid derivative or a specific amino acid with its amino or carboxyl end blocked. More than half of them were annotated only once in the whole UniProtKB, while the most annotated case (blocked amino end (Ala)) had only 30 instances of it.

Among the 27 PTMs annotated in UniProtKB as protein core, the vast majority is D‐ and didehydro‐ amino acids. From these, only nine were annotated at least 10x in the whole database. When analyzing them, only the three most frequent ones had a positive score, indicative of a 3D structure association (see also Table 2). They were 2,3‐didehydroalanine (Ser), 1‐thioglycine and 2,3‐didehydroalanine (Cys) with 292, 86 and 35 instances annotated, respectively.

Another PTM annotated as protein core in the position of the modification field in UniProtKB is 3’‐*nitrotyrosine*. This PTM only had three instances annotated falling out of our cut‐off threshold for the analysis. But given that this PTM was not one of the common D‐ or didehydro‐ amino acids from the list, it was clear that 3′‐nitrotyrosine, which relates to the existing highly annotated nitrated tyrosine (labeled by UniProtKB as anywhere) with 352 instances, also shown in our Fig. 5, should be just a matter of time to be reconsidered in the UniProtKB controlled vocabulary list.

Analysis of *2,3‐didehydroalanine* (Ser), as the most annotated case in protein core (292 instances, score = 0.75, accessibility = 0.19 ± 0.05), showed that this modification was found in different ammonia‐lyases. For instance, 4‐methylidene‐imidazole‐5‐one (MIO) is produced autocatalytically by cyclization and dehydration of residues Ala‐Ser‐Gly 131.

Interestingly, all instances annotated with “glycyl adenylate; alternate” (commented in MOD_RES N‐ and C‐terminal PTMs with low relative residue accessibility) were annotated as “1‐thioglycine; alternate”, as well. This is because they are part of a two‐step process where a C‐terminal glycine is first activated as glycyl adenylate which is then replaced by thiocarboxylation. As we previously explained for glycyl adenylate, its C‐terminal is non‐accessible due to complex formation (Supporting Information Fig. 8(e) and (f)). This is an example where classification of PTMs in regard to their localization is work in progress in the ptmlist.txt of UniProtKB. 1‐thioglycine was the second most annotated UniProtKB protein core PTM (86 instances) and our analysis pointed out to its high accessibility for the reasons already described (score = 0.91, accessibility = 0.88 ± 0.64).

*2,3‐didehydroalanine* (Cys) was the third most occurring PTM and its instances were mapped to thiamine thiazole synthases of fungi and plants. Structural elucidation of this suicide enzyme 132, 133 indicates that the modified residue is accessible in the structure monomer but buried upon complex formation of its homo 8‐mer biological assembly.

## 4 Discussion

The influence of PTMs on the structure and function of proteins has become an increasingly important research topic since data about PTMs is growing in availability. Resources such as RESID 134 and “ptmlist.txt” from UniProtKB 4 provide comprehensive lists of known residue modifications. There are also databases for access to information about modified residues observed in 3D structures of proteins such as PDBsite 135, 136, AMASS 137 and PTM‐SD 138. Software tools for analyzing the role of modified residues within the protein's 3D structure in molecular simulations are also available 139, 140, 141. Unrivalled, UniProtKB allows access to the largest database of diverse PTMs mapped onto protein sequences. The number of different PTMs that could be seen in 3D structures is much smaller than the >300 various PTMs described in UniProtKB. Notably, PTM‐SD reports only 21 PTM types 138 and, obviously, many PTMs render the respective modified proteins non‐suitable for crystallization. During the last decade alone, the number of known PTMs has been increased by several hundred new types and large‐scale proteomic methods have also resulted in a 5–10 fold increase in reported instances for each PTM.

In this work, we explored the question whether there are sets of PTMs in the protein databases that would occur predominantly in regions of tertiary structure or outside of them. The starting point was the insight that, for a PTM to be introduced into a substrate protein by a posttranslationally modifying enzyme, the sequence stretch carrying the residue to be modified, at some stage, must be accessible to the enzyme and be able to conformationally adapt to the setup in the catalytic crevice. This accessibility and flexibility criterion considerably restricts the type of sequence segments that can typically become targets of PTMs 10, 11. As studies for specific PTM types such as GPI lipid anchoring 12, 38, 142, 143, 144, 145, myristoylation 14, 37, 146, prenylation 15, 46, phosphorylation 17, 20 as well as the case of the PTS1 signal for peroxisomal import 16, 147, 148 have shown, the sequence segment of the substrate protein that is engulfed by the enzyme's binding site has to be surrounded by a linker‐type region with a trend towards polar residues with small side‐chain volume and flexible backbone. The accessibility requirement appears to be incompatible with the residue to be modified being buried in the tertiary structure.

The accessibility and flexibility requirement has significance not only for the creation of the PTM by the posttranslationally modifying enzyme. For the modified residue to change the biological properties of the substrate protein, accessibility and flexibility of the sequence segment with the modified residue is also an advantage since it enhances the possibility for recognition by other proteins including enzymes that, at a later stage, might remove the PTM. For example, this possibility was discussed for the case of tyrosine nitration 116, 149. But the accessibility of the modified residue is not an absolute necessity for function. Of course, the biological effect of a PTM can also be mediated in a more indirect manner by facilitating conformational changes, affecting inter‐domain and inter‐subunit interactions, etc., which in turn might affect the capacity for further posttranslational modifications. Phosphorylation has been shown to drive conformational changes in the sarcoplasmic reticulum ATPase and in Rap1b 150, 151. Experimental and computational examples for PTMs that can cause conformational changes over distance in an allosteric manner have been described in the literature 152, 153, 154 and a webserver is available to test further cases when the 3D structure coordinates are known 155.

As a matter of fact, the functional significance of many PTMs is currently not known. For example, the role of many structural variants of N‐glycans 156 remains to be established. Therefore, great research efforts are focused on problem of PTM functions and significant progress has been made recently with regard to selected PTMs such as lysine acetylation 157, more recently discovered modifications succinylation, malonylation and glutarylation 158, or extension of arginine methylation to non‐histone proteins 159. Even mutation experiments that suppress the PTM in a substrate protein are sometimes inconclusive since the absence of the PTM is not associated with a phenotypic change, at least in the conditions of those studies. For example in the case of lipidation, the observation might be associated with the fact that membrane location is not only promoted by the lipid anchor but also by interaction with an anyhow membrane‐bound complex 2, 46, 160, 161, 162. Yet, it appears early to negate any biological role for such PTMs. For example, the anchor's effect might be relevant only in unknown extreme conditions or it serves as an evolutionary stabilizer in the case of mutations weakening the interaction complex.

Here, we represent the first in‐depth study to which extent UniProtKB annotated PTMs are related to known 3D structures of proteins and how this might affect the mechanism of their genesis. In previous reports, the question of the structural environment of PTM sites was analyzed on more limited datasets. Pang et al. 106 investigated the structural surroundings (based on sequence information and predictors for disorder, accessibility and linker properties) of 44 PTMs only annotated under MOD_RES and O‐GlcNAc (lipids were excluded, while in our study, we excluded glycosylation sites). Interestingly, Pang et al. also reported the PTMs phosphohistidine and 4‐aspartylphosphate in protein cores and mention their involvement in bacterial two‐component systems. Our study covers many more PTMs and added actual 3D structure information to the analysis. Xie et al. 139 and Gao and Xu 87 found significant correlations between certain PTM types and their occurrence within disordered regions.

As in any large‐scale study, the conclusions in this work depend on the quality and the completeness of the input data to a considerable extent. First of all, we think that not all actually structured regions are represented by sequence‐similar examples of 3D structures in the PDB and a future repetition of this work might find a more complete sequence coverage with then known tertiary structures. Thus, the 3D structure scores should be rather considered lower estimates. Nevertheless, it is striking that there are very few examples of PTMs that mostly occur in structured regions. Also, we have to assume that the PTMs annotated in the UniProtKB are correctly identified in the sequences but they might be mispredicted by mass‐spectrometry techniques or software tools 163, 164, 165. Further, we assume that the protein occurs in the biological context as full‐length protein when alternative splicing, cleavage of targeting peptides and other types of protein processing might actually alter the sequence and make sites that were previously thought non‐accessible actually accessible and *vice versa*.

N‐ and C‐ termini of proteins are well known to be related to flexible regions in many cases and for having higher propensity to being disordered. The nature of this flexibility has been shown to be important for their functionality as well 23. We find in our study that, except for the case of serine‐to‐pyruvic acid self‐processing, all N‐ and C‐terminal modification types studied benefit from the enhanced accessibility of the termini and are most likely canonical, enzyme‐generated PTMs. Similarly, all lipid anchor modifications analyzed (with the possible exception of few acylation cases with reactive acyl‐CoA species at internal protein sequence positions where further research might be required) are canonical, enzyme generated PTMs.

The more complex cases are those summarized in the two sections about MOD_RES annotated PTMs with low accessibility. In the cases where no mechanism involving an outside enzyme could be drawn for explanation, most of the cases could be rationalized either as autocatalytic/self‐processing, PTMs before/during folding or with temporary unfolding or as reaction with small reactants diffusing into the 3D structure. The interpretation gets difficult in cases of relatively rare PTMs and, for a list of PTMs, only further research can clarify the matter. Among others, we would include the following PTMs into this category: Aspartyl aldehyde, tyrosine nitration, S‐nitrosocysteine, N6‐(retinylidene)lysine.

The relationship between regions of structural disorder and PTM sites is certainly an interesting topic. Unfortunately, the DisProt database 31 is still small to make an overall impact for a large‐scale study as this one. Yet for some PTMs with rare occurrences, the disorder information from DisProt is critical as in the cases of the PTMs tyrosine nitration and S‐nitrosocysteine (Supporting Information Fig. 12), both with 1 and 3 entries, respectively, matching a DisProt disordered region. For instance, the 3 S‐nitrosocysteine entries matching DisProt were associated to three different structures in our analysis, with accessibility ranging from 0.01 to 0.84. The buried example, the C‐terminal activation domain of hypoxia‐inducible factor 1‐alpha (Q16665), has been identified as an intrinsically disordered domain that remains relatively extended upon binding 166, indicating, perhaps, that PTMs involving reactive small molecules and that fall out of the canonical PTM type extensively presented here, could occur more often in intrinsically disordered proteins, which consequently would additionally present a wide range of accessibility upon structural elucidation. Another example has been the transcription factor p65 (Q04207), which we associated to a highly exposed site (0.84). Disorder has been shown to be an important factor for its functionality 167.

For lipid modifications (see Supporting Information. Fig. 13), the DisProt analysis only found overlap with the PTMs N‐palmitoyl cysteine, S‐diacylglycerol cysteine and N‐myristoyl glycine for N‐terminal, as well as S‐palmitoyl cysteine and S‐(15‐deoxy‐Delta12,14‐prostaglandin J2‐9‐yl)cysteine for anywhere. In all the cases, DisProt complemented what has been observed, with the exception of S‐(15‐deoxy‐Delta12,14‐prostaglandin for which we had a positive 3D structure score of 0.4 (Fig. 3). Still, the entry that overlapped with DisProt was also not directly related to a structure, contributing to the “negative” part of the score.

As the trend in the results point to the overwhelming majority of PTMs being embedded in conformationally flexible sequence regions with many small and polar residues, we find that the amino acid compositional restrictions are not strong enough to have these sequences be hit by tools that are designed to recognize intrinsically disordered regions in all cases though many are for certain PTMs. Thus, the criteria implemented in these tools appear too crude to capture the integral sequence properties of these regions in a variety of cases. This result is in line with the observations in the literature 90, 168 (see Supporting Information Tables 3 and 4 with DisProt and IUpred results for all lipid and MOD_RES PTMs studied, respectively).

Of course, the ultimate goal is to predict all kinds of PTM sites directly from the substrate protein with enzyme‐specific prediction tools. This work shows that, in addition to predictor components that recognize the position‐specific amino acid type preferences next to the site to be modified, the more integral properties of the surrounding sequence stretches at either side of the PTM site (namely, the requirement for a trend towards flexibility, polarity and small side chain volume) provide valuable restrictions for the sequence search space 15, 37, 38, 142, 145, 147. Similarly, this seems important for protease cleavage sites in substrate proteins 22, 169, 170, 171, 172 and, apparently, for many other PTMs.

The great challenge for today's life science research is to link specific genomic features and sequences with phenotypic properties via biomolecular mechanisms 6, 173, 174. Special attention is needed for the vast arrays of functionally uncharacterized genome sections in human and most other organisms. With regard to protein‐coding regions, the annotation status of non‐globular segments is especially unsatisfactory 6, 175, 176, 177, 178. Since many PTMs are harbored in such segments, the research dedicated to elucidate the functional role of PTMs promises to have important impact.

### 4.1 Conclusions

In summary, many, if not the majority of protein posttranslational modifications (PTMs) are the result of an enzymatic process. It involves the recognition by the posttranslationally modifying enzyme of a sequence motif in the substrate protein containing the residue(s) modified; thus, the catalytic cleft of the enzyme has to engulf the residue(s) to be modified at some reaction stage. This residue accessibility condition principally limits the range where PTMs can occur in the protein sequence and has implications on the prediction of such motifs from sequence aiding in protein functional annotation.

We have demonstrated with quantified arguments that essentially all N‐ and C‐terminal PTMs as well as the overwhelming majority of PTMs localized elsewhere in the sequence are found in sequence environments accessible to outside modifying enzymes, mostly even in unstructured or accessible/flexible structured regions. Few PTMs that were found truly buried in 3D structures have been listed, being mostly involved in autocatalysis/self‐processing, reaction with diffusing small reactants and in reaction events before/during folding and temporary unfolding. A few, currently rare cases of PTMs require further research.

## Supporting information

As a service to our authors and readers, this journal provides supporting information supplied by the authors. Such materials are peer reviewed and may be re‐organized for online delivery, but are not copy‐edited or typeset. Technical support issues arising from supporting information (other than missing files) should be addressed to the authors.

Figure 1. Structural overlap of 11 chains from the following PDB structures
Figure 2.Structural superposition of 2NGR:A, 2DFK:B, 1A4R:A and 4F38:A.
Figure 3. PDB structure 4DM9 [48] of ubiquitin C‐terminal hydrolase L1 (UCHL1).
Figure 4. Crystal structure of *Xenopus laevis* Wnt8 (in orange) in complex with the cysteine‐rich domain of Frizzled
Figure 5. Electron cryo‐microscopy of Chikungunya virus displaying the glycoprotein E1 in red and its transmembrane helix in blue.
Figure 6.Blast alignment of human P‐selectin to the PDB structure of apolipoprotein‐H with the modified cysteine 807 highlighted in red.
Figure 7. Structures of two non‐specific lipid‐transfer proteins from wheat (left) and barley (right).
Figure 8. Examples of significant structural hits of C‐terminal PTMs with positive score, but with low accessibility.
Figure 9. Number of PTM instances of (*a*) phosphoserine, (*b*) phosphothreonine, (*c*) phosphotyrosine and (*d*) phosphohistidine
Figure 10.Structures of elongation factor P (*a*) 3A5Z:B [43] and (*b*) 3TRE:A (Cheung et al., to be published).
Figure 11. Structures of 1B33 and 1ON7 superimposed to highlight the methylated (red) and unmethylated (yellow) asparagine residues of 1B33 and 1ON7, respectively.
Figure 12. Percentage of lipid PTM sites mapped to a DisProt region (red), predicted as disordered by IUPred (blue) or both (green) in regard to the total number of instances annotated (black curve).
Figure 13.Percentage of MOD_RES PTM sites that fulfilled the same requirements of those displayed in Fig. 5 of the paper, mapped to a DisProt region (red), predicted as disordered by IUPred (blue) or both (green) in regard to the total number of instances annotated (black curve).

Table 1. Summary of PTM analysis annotated under MOD_RES with a negative score and low accessibility.
Table 2. Summary of PTM analysis annotated under MOD_RES mostly with a positive score and low accessibility.
Table 3. Summary of lipid PTM sites hitting DisProt‐ or IUpred‐predicted regions
