# Supplementary material for: Single‐residue posttranslational modification sites at the N‐terminus, C‐terminus or in‐between: To be or not to be exposed for enzyme access
Source: Proteomics. 2015 Jul 14;15(14):2525–46. doi: 10.1002/pmic.201400633 (PMC4745020; doi:10.1002/pmic.201400633)
Supplement: Supplementary file 1 — Figure 1. Structural overlap of 11 chains from the following PDB structures Figure 2.Structural superposition of 2NGR:A, 2DFK:B, 1A4R:A and 4F38:A. Figure 3. PDB structure 4DM9 [48] of ubiquitin C‐terminal hydrolase L1 (UCHL1). Figure 4. Crystal structure of Xenopus laevis Wnt8 (in orange) in complex with the cysteine‐rich domain of Frizzled Figure 5. Electron cryo‐microscopy of Chikungunya virus displaying the glycoprotein E1 in red and its transmembrane helix in blue. Figure 6.Blast alignment of human P‐selectin to the PDB structure of apolipoprotein‐H with the modified cysteine 807 highlighted in red. Figure 7. Structures of two non‐specific lipid‐transfer proteins from wheat (left) and barley (right). Figure 8. Examples of significant structural hits of C‐terminal PTMs with positive score, but with low accessibility. Figure 9. Number of PTM instances of (a) phosphoserine, (b) phosphothreonine, (c) phosphotyrosine and (d) phosphohistidine Figure 10.Structures of elongation factor P (a) 3A5Z:B [43] and (b) 3TRE:A (Cheung et al., to be published). Figure 11. Structures of 1B33 and 1ON7 superimposed to highlight the methylated (red) and unmethylated (yellow) asparagine residues of 1B33 and 1ON7, respectively. Figure 12. Percentage of lipid PTM sites mapped to a DisProt region (red), predicted as disordered by IUPred (blue) or both (green) in regard to the total number of instances annotated (black curve). Figure 13.Percentage of MOD_RES PTM sites that fulfilled the same requirements of those displayed in Fig. 5 of the paper, mapped to a DisProt region (red), predicted as disordered by IUPred (blue) or both (green) in regard to the total number of instances annotated (black curve). [file PMIC-15-2525-s001.pptx]

## Slide 1
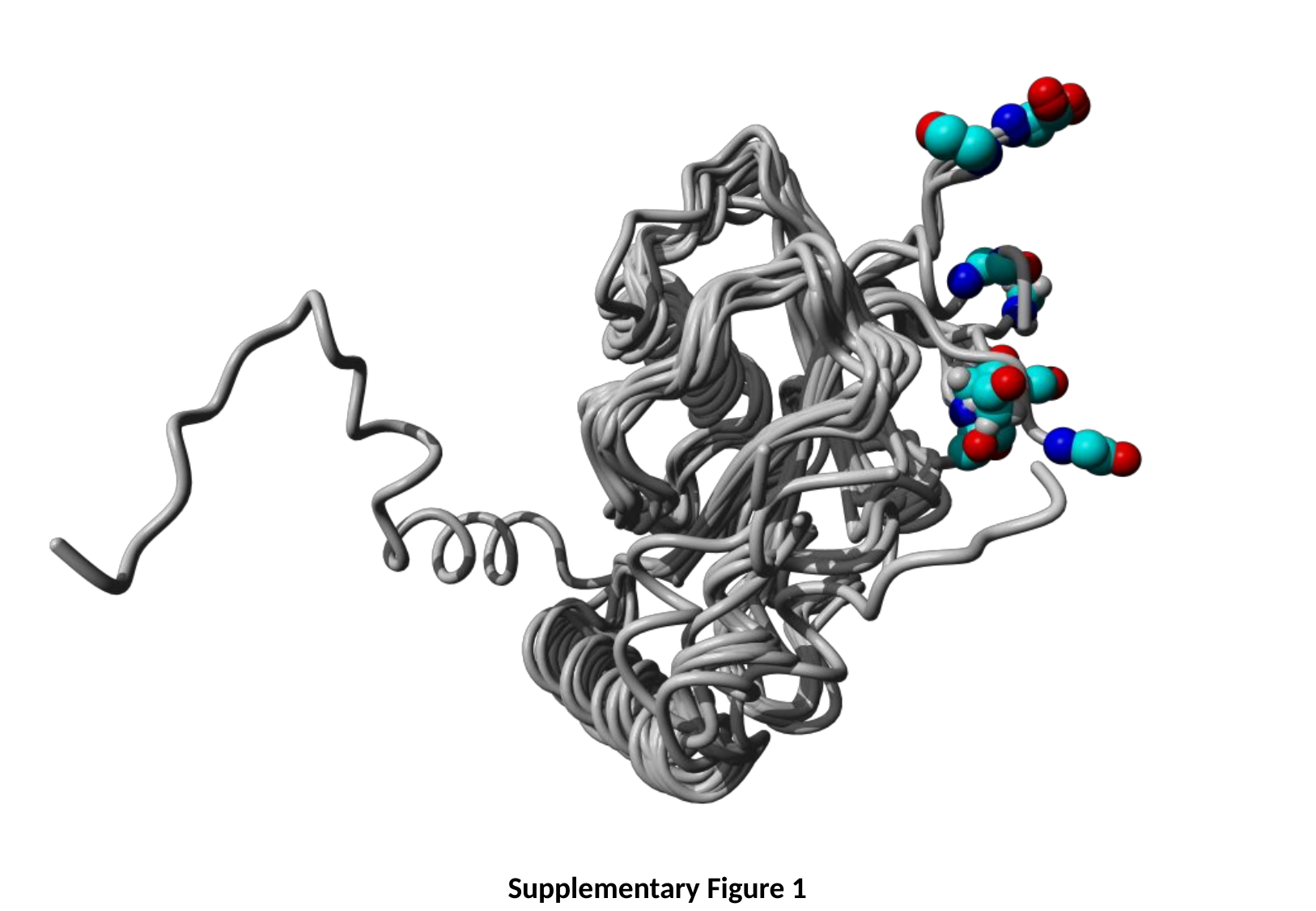

Supplementary Figure 1

## Slide 2
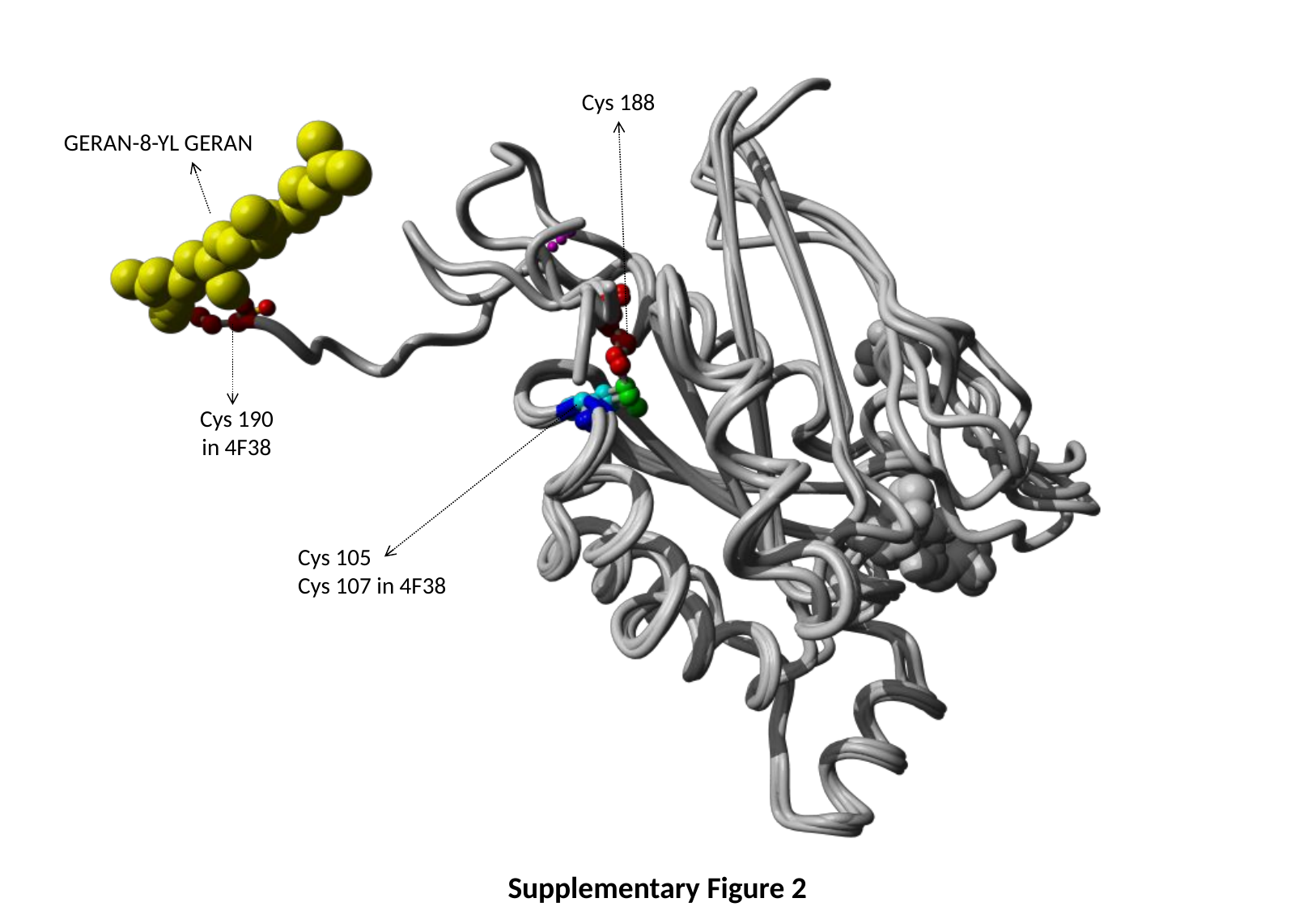

Cys 188
GERAN-8-YL GERAN
Cys 190
in 4F38
Cys 105
Cys 107 in 4F38
Supplementary Figure 2

## Slide 3
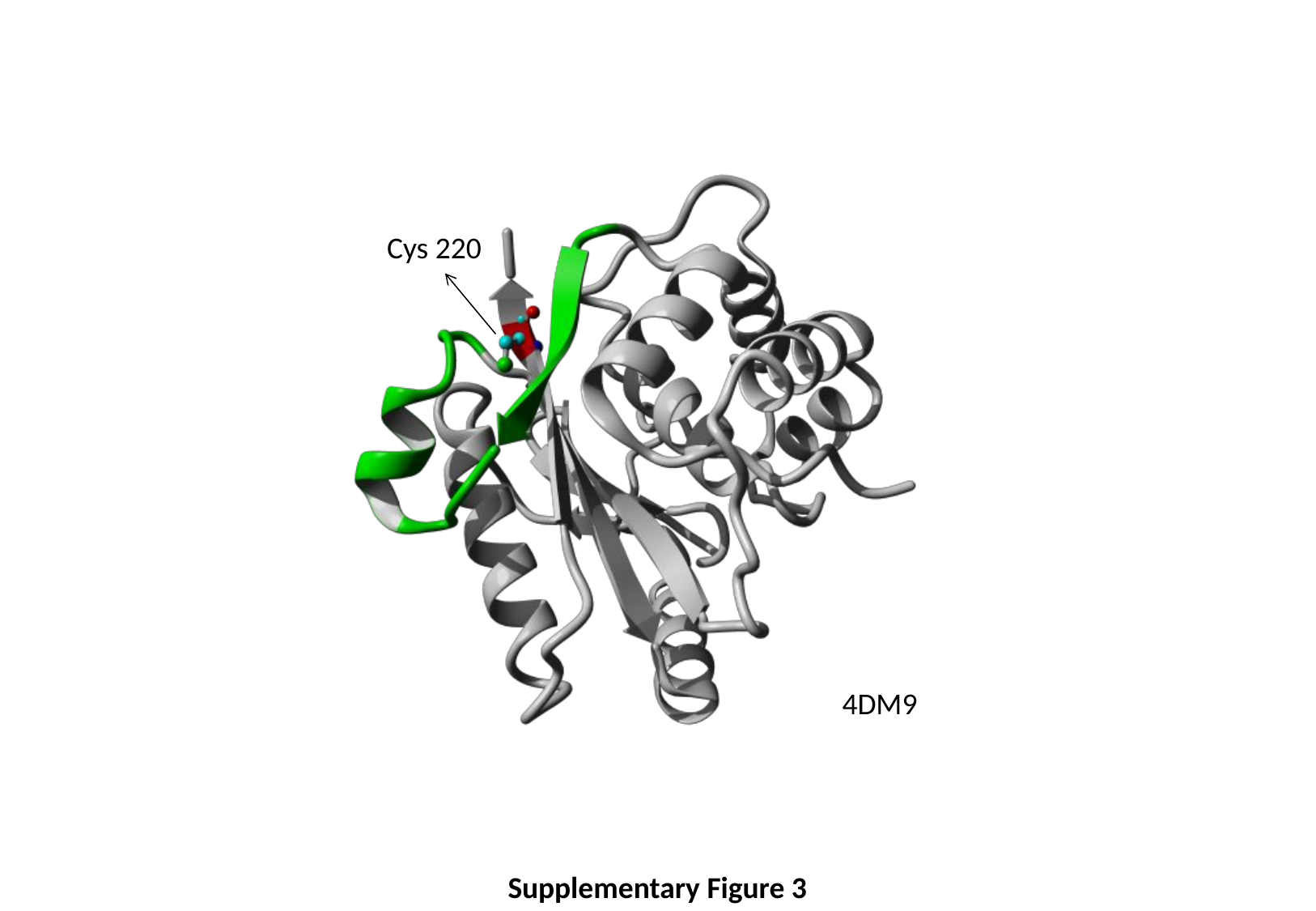

Cys 220
4DM9
Supplementary Figure 3

## Slide 4
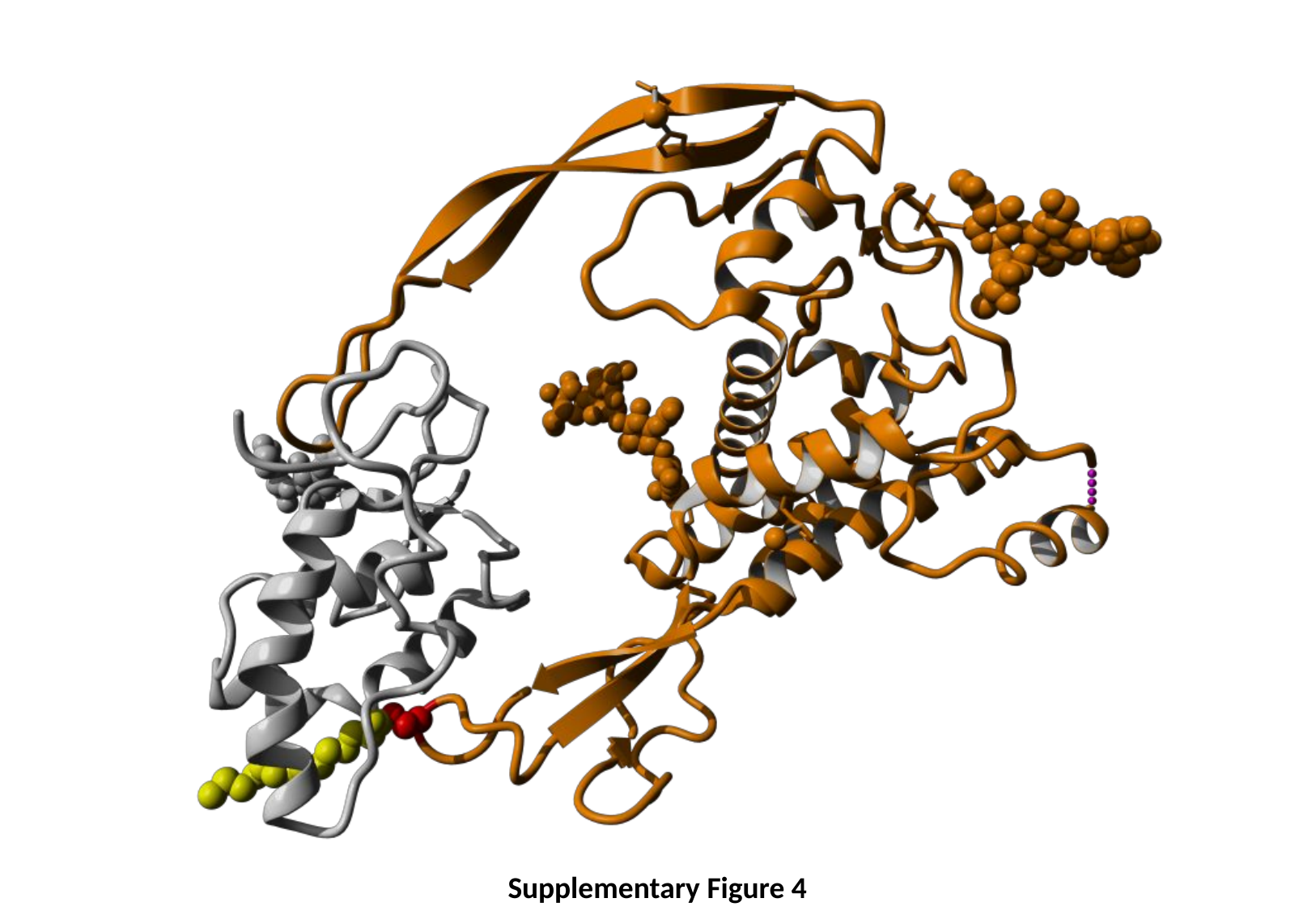

Supplementary Figure 4

## Slide 5
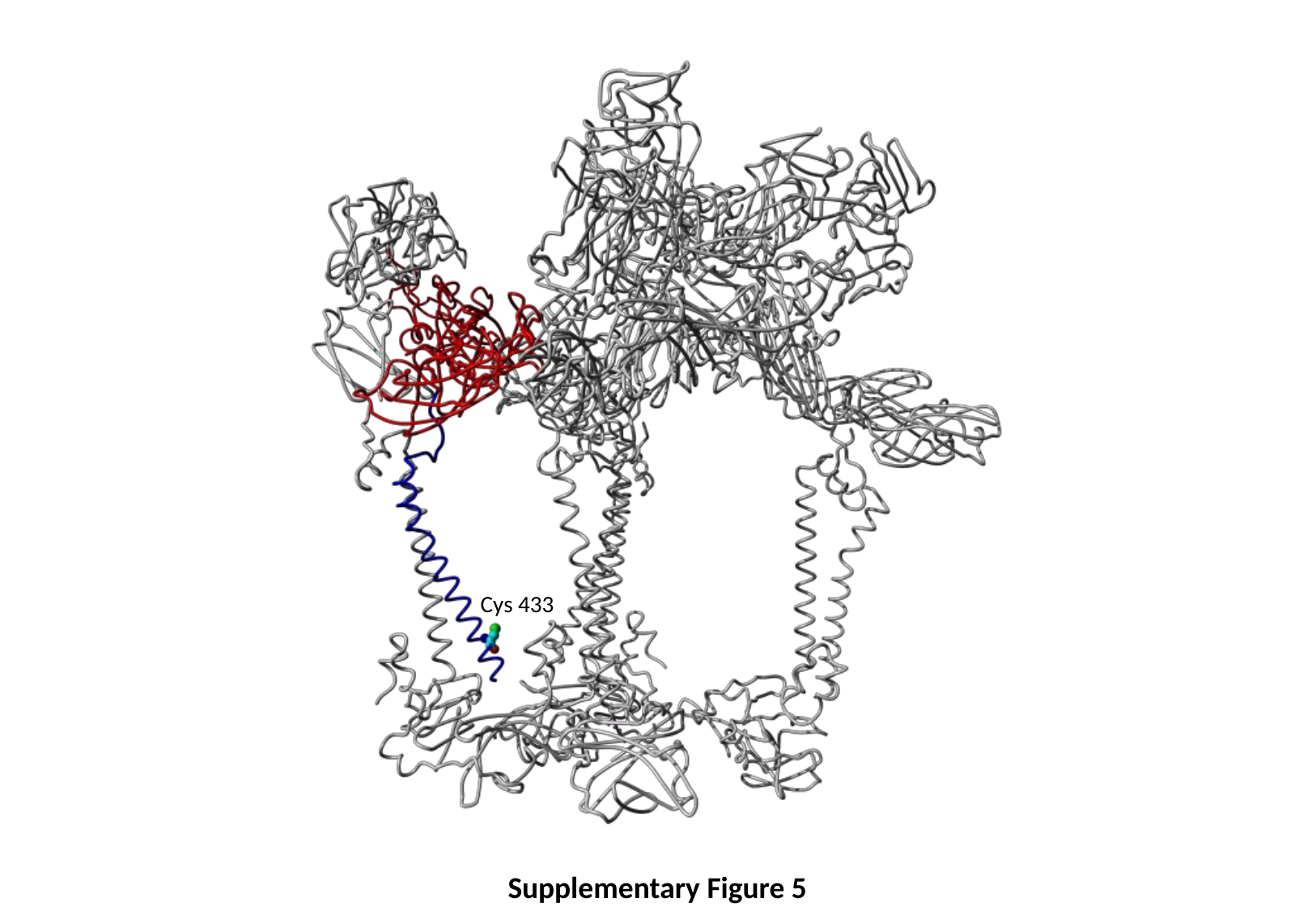

Cys 433
Supplementary Figure 5

## Slide 6
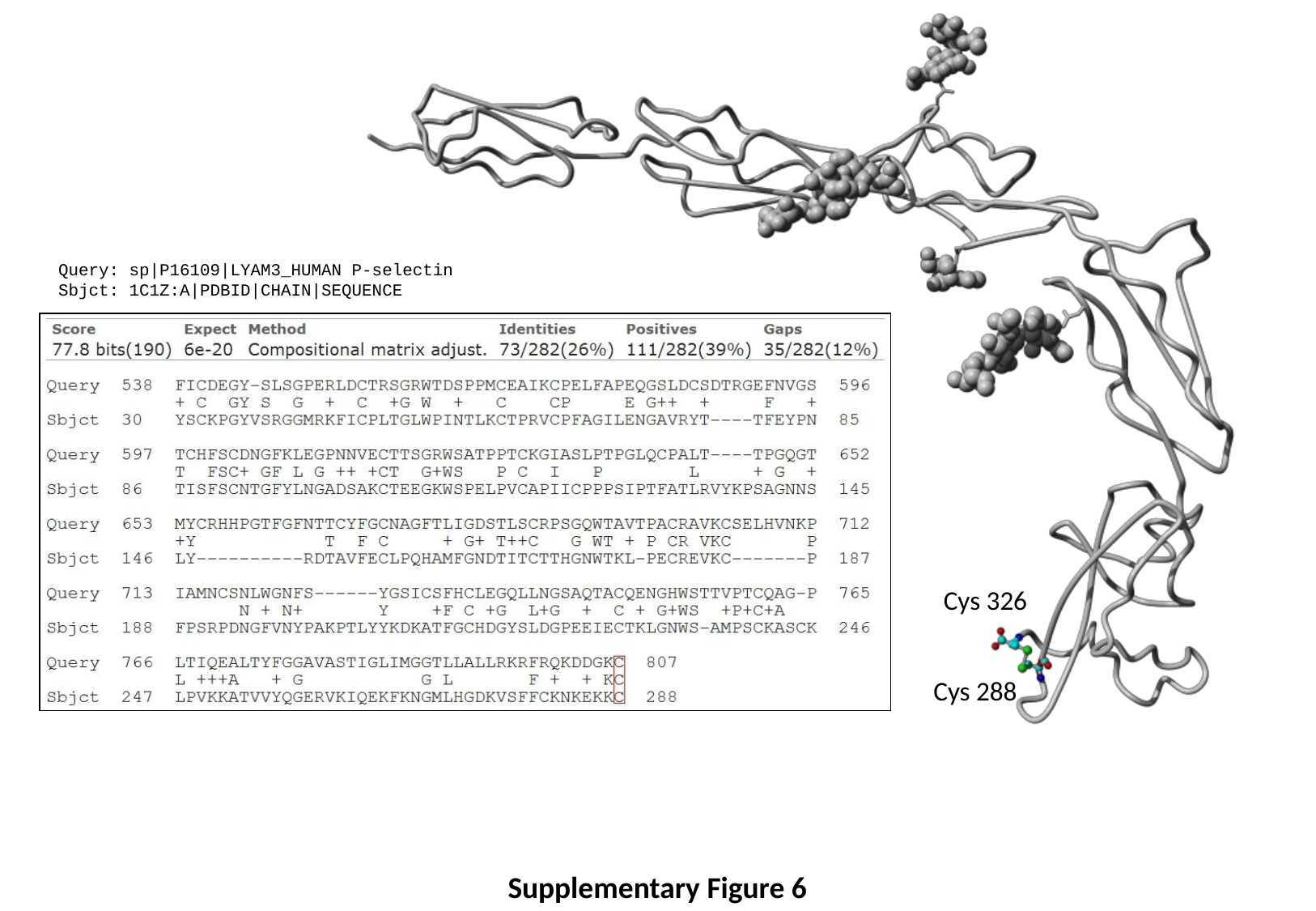

Query: sp|P16109|LYAM3_HUMAN P-selectin
Sbjct: 1C1Z:A|PDBID|CHAIN|SEQUENCE
Cys 326
Cys 288
Supplementary Figure 6

## Slide 7
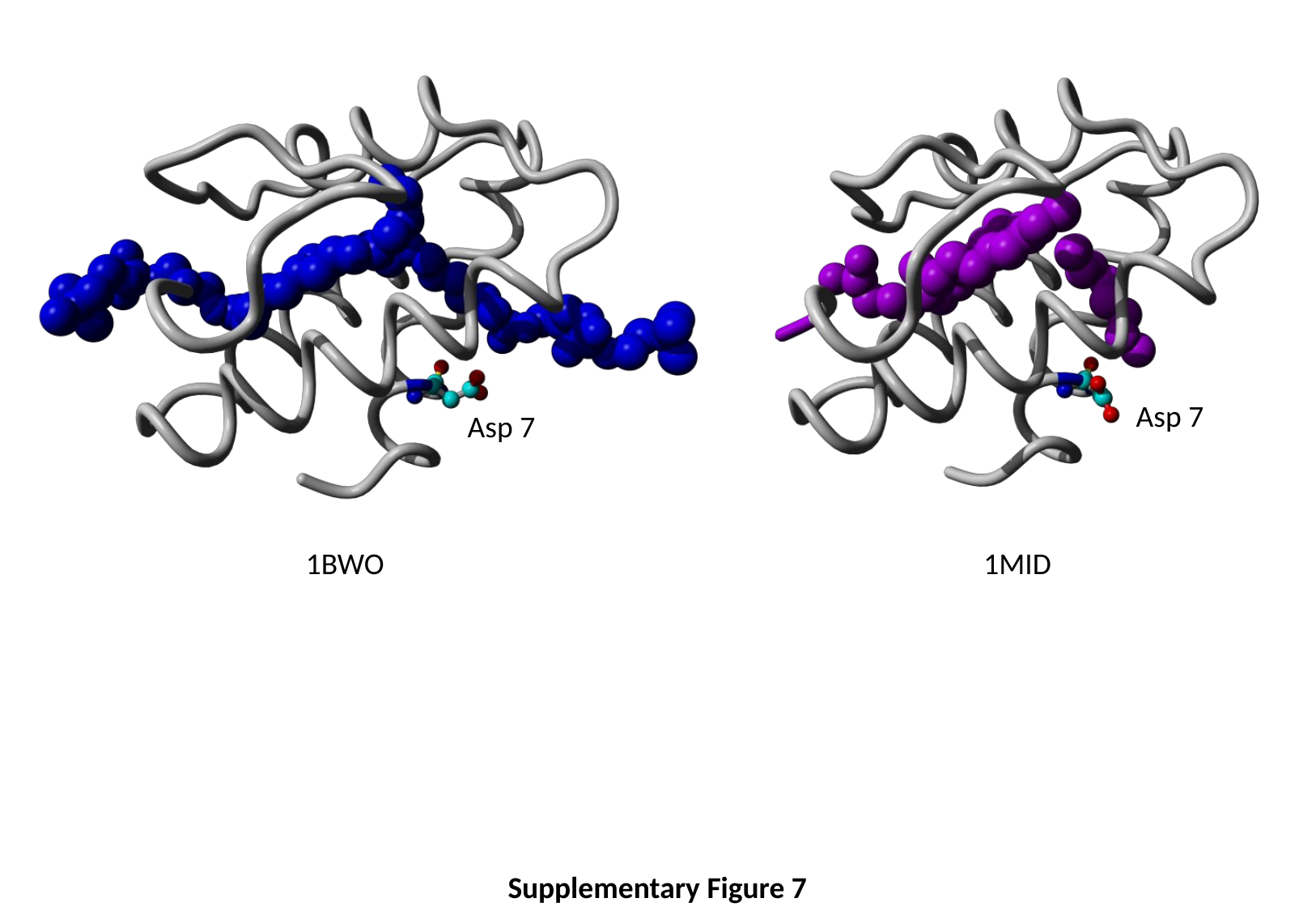

Asp 7
Asp 7
1BWO
1MID
Supplementary Figure 7

## Slide 8
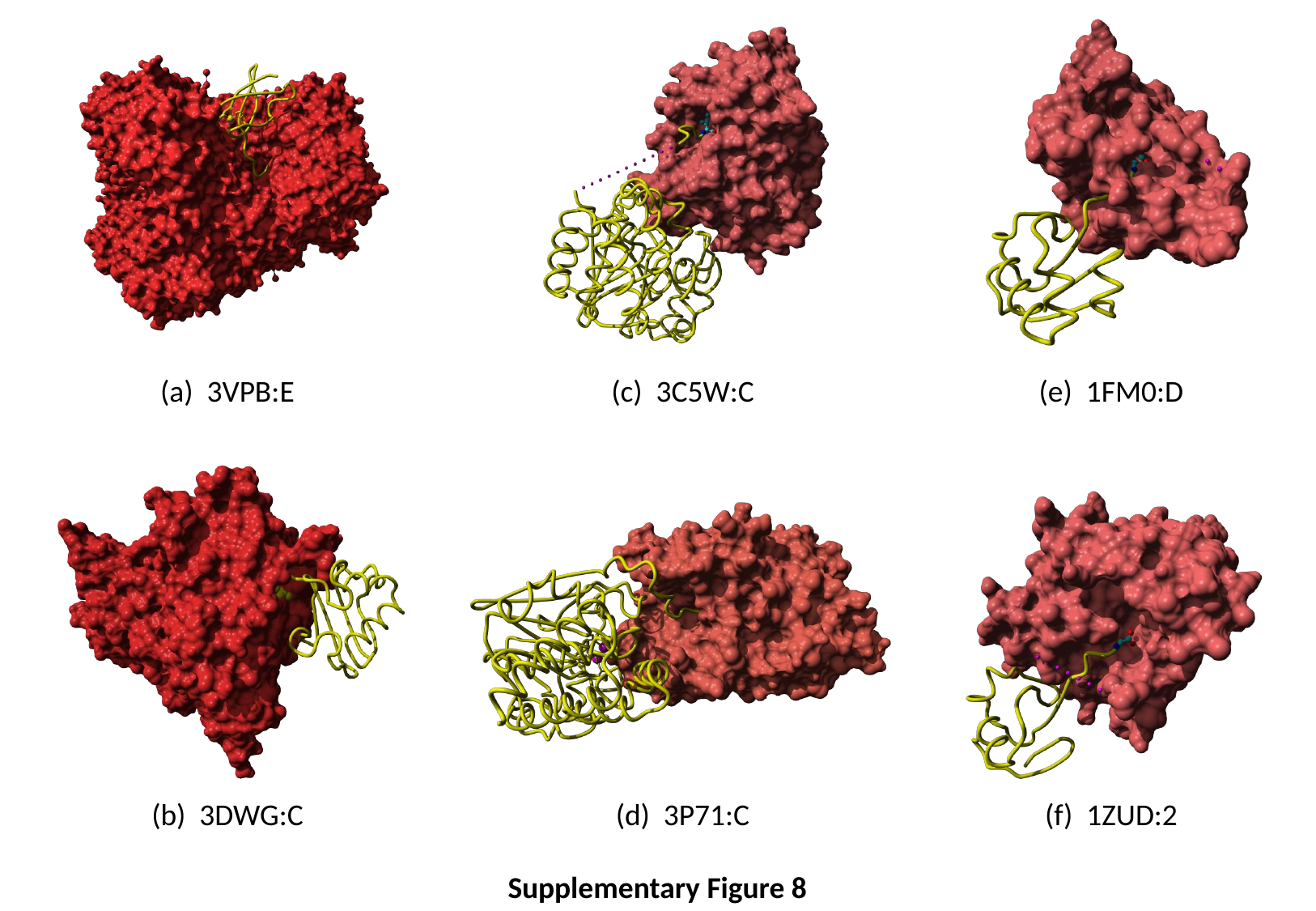

(a) 3VPB:E
(c) 3C5W:C
(e) 1FM0:D
(b) 3DWG:C
(d) 3P71:C
(f) 1ZUD:2
Supplementary Figure 8

## Slide 9
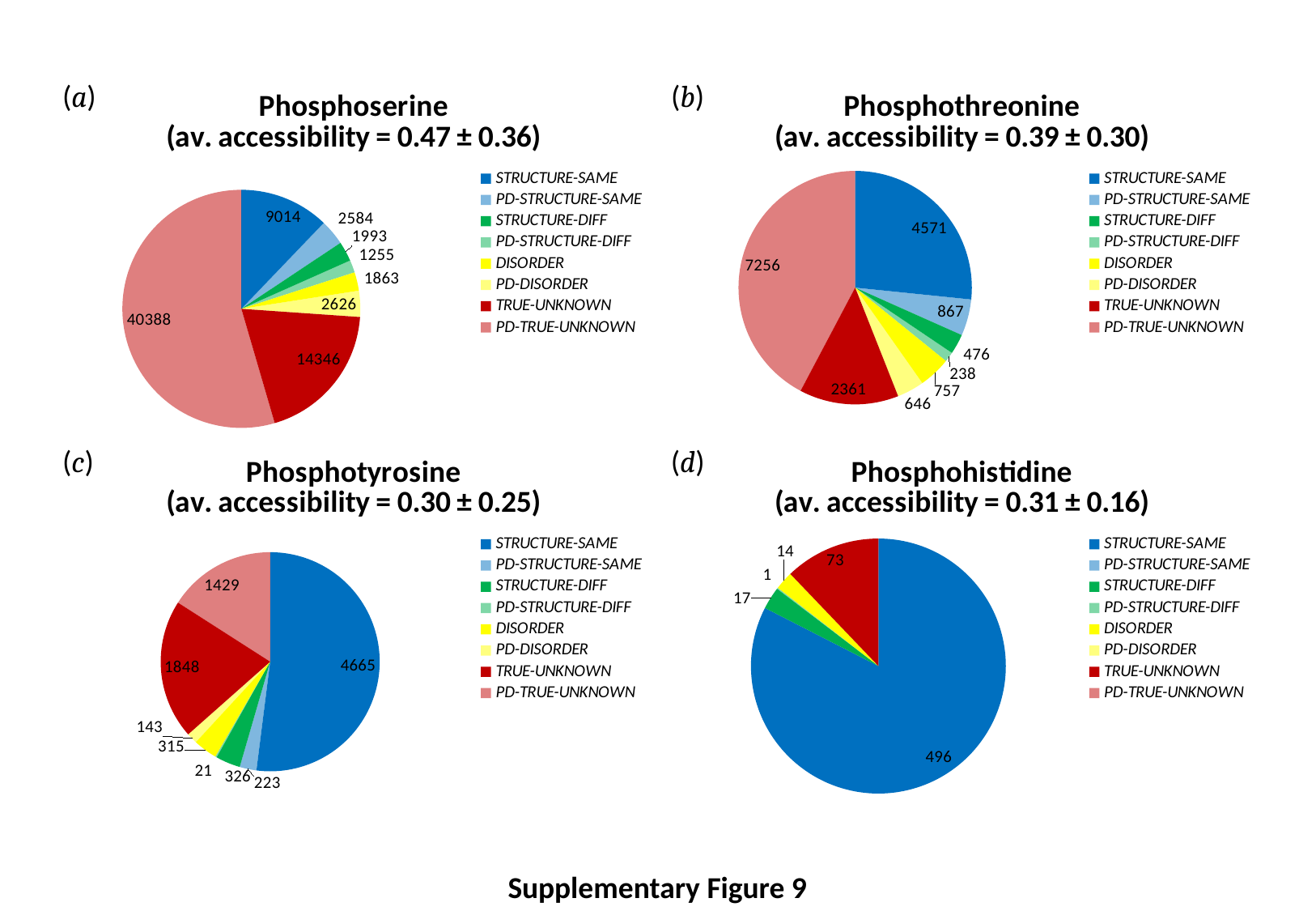

### Chart: Phosphoserine
(av. accessibility = 0.47 ± 0.36)
| Category | Phosphoserine |
|---|---|
| STRUCTURE-SAME | 9014.0 |
| PD-STRUCTURE-SAME | 2584.0 |
| STRUCTURE-DIFF | 1993.0 |
| PD-STRUCTURE-DIFF | 1255.0 |
| DISORDER | 1863.0 |
| PD-DISORDER | 2626.0 |
| TRUE-UNKNOWN | 14346.0 |
| PD-TRUE-UNKNOWN | 40388.0 |(a)
### Chart: Phosphothreonine
(av. accessibility = 0.39 ± 0.30)
| Category | Phosphothreonine |
|---|---|
| STRUCTURE-SAME | 4571.0 |
| PD-STRUCTURE-SAME | 867.0 |
| STRUCTURE-DIFF | 476.0 |
| PD-STRUCTURE-DIFF | 238.0 |
| DISORDER | 757.0 |
| PD-DISORDER | 646.0 |
| TRUE-UNKNOWN | 2361.0 |
| PD-TRUE-UNKNOWN | 7256.0 |(b)
### Chart: Phosphotyrosine
(av. accessibility = 0.30 ± 0.25)
| Category | Phosphotyrosine |
|---|---|
| STRUCTURE-SAME | 4665.0 |
| PD-STRUCTURE-SAME | 223.0 |
| STRUCTURE-DIFF | 326.0 |
| PD-STRUCTURE-DIFF | 21.0 |
| DISORDER | 315.0 |
| PD-DISORDER | 143.0 |
| TRUE-UNKNOWN | 1848.0 |
| PD-TRUE-UNKNOWN | 1429.0 |(c)
### Chart: Phosphohistidine
(av. accessibility = 0.31 ± 0.16)
| Category | Phosphohistidine |
|---|---|
| STRUCTURE-SAME | 496.0 |
| PD-STRUCTURE-SAME | 0.0 |
| STRUCTURE-DIFF | 17.0 |
| PD-STRUCTURE-DIFF | 1.0 |
| DISORDER | 14.0 |
| PD-DISORDER | 0.0 |
| TRUE-UNKNOWN | 73.0 |
| PD-TRUE-UNKNOWN | 0.0 |(d)
Supplementary Figure 9

## Slide 10
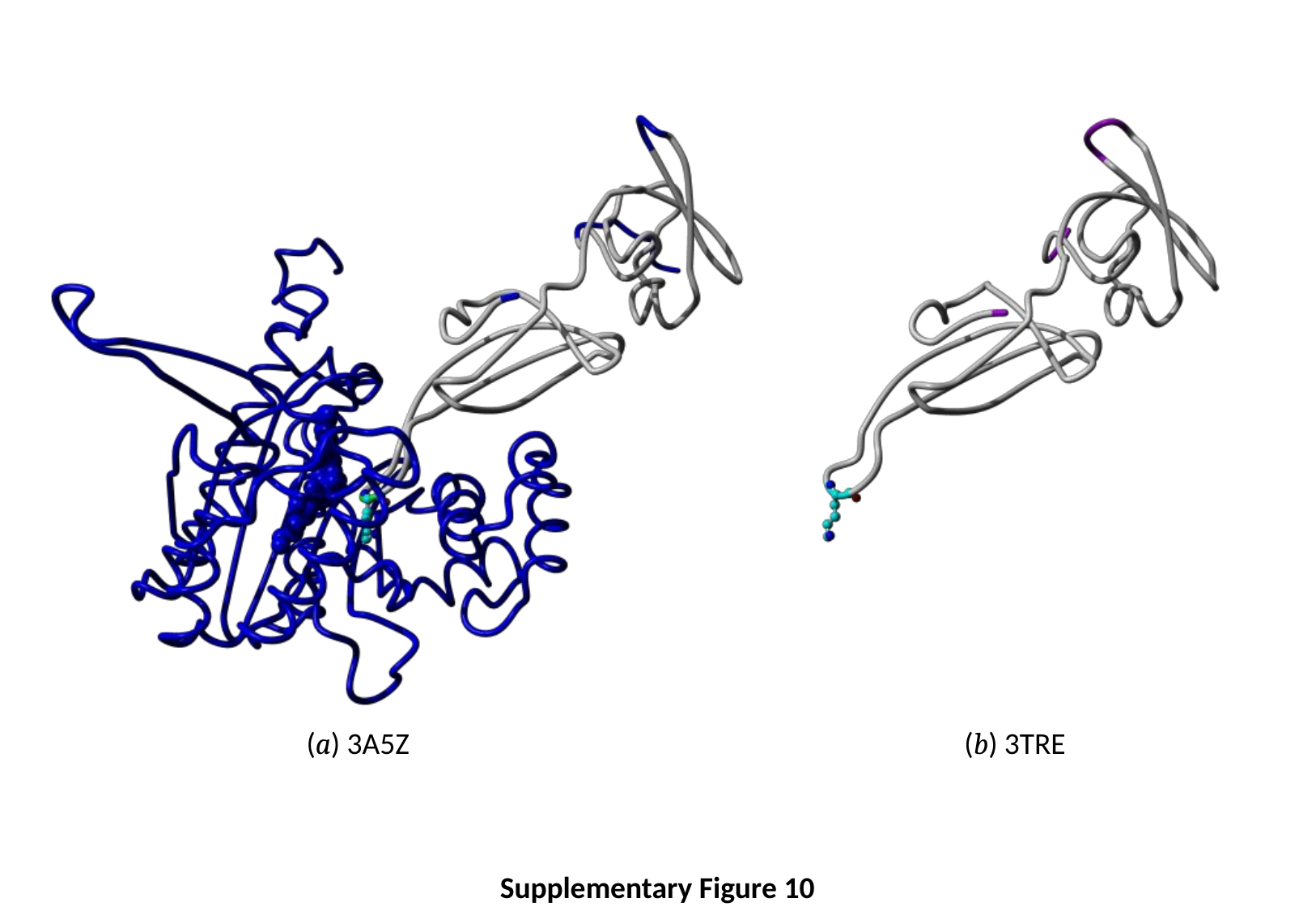

(a) 3A5Z
(b) 3TRE
Supplementary Figure 10

## Slide 11
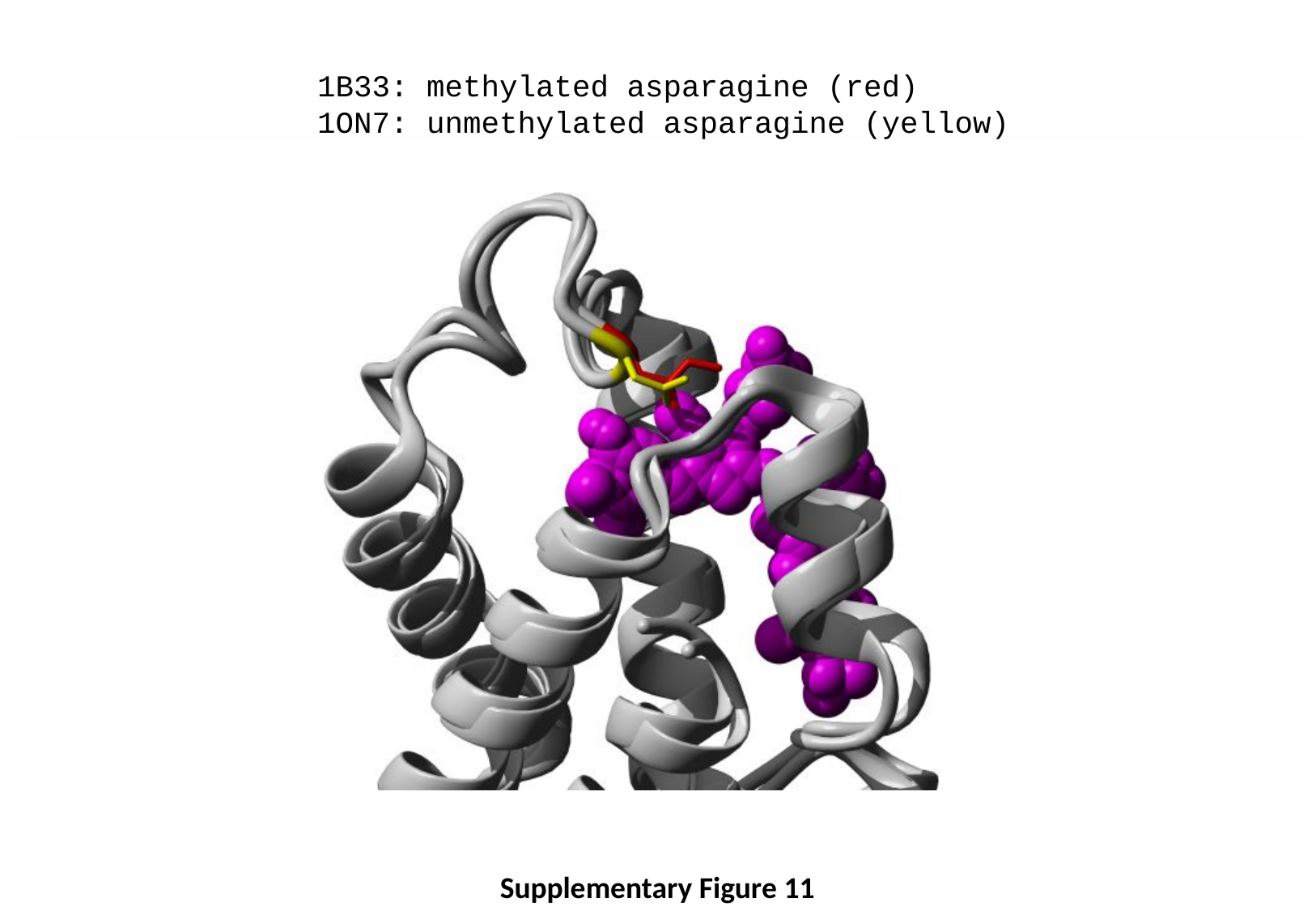

1B33: methylated asparagine (red)
1ON7: unmethylated asparagine (yellow)
Supplementary Figure 11

## Slide 12
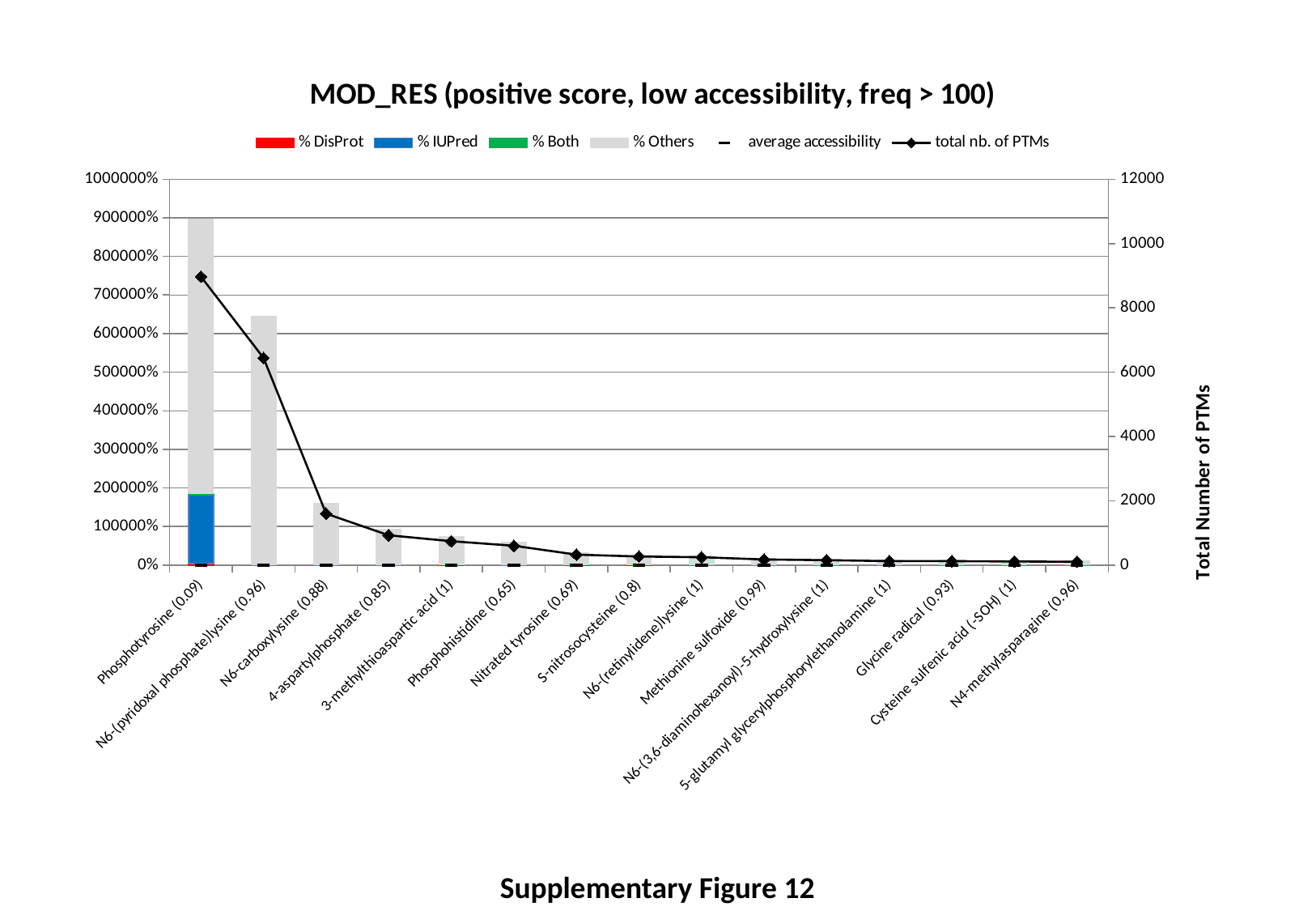

### Chart: MOD_RES (positive score, low accessibility, freq > 100)
| Category | % DisProt | % IUPred | % Both | % Others | average accessibility | total nb. of PTMs |
|---|---|---|---|---|---|---|
| Phosphotyrosine (0.09) | 31.0 | 1795.0 | 21.0 | 7123.0 | 0.3 | 8970.0 |
| N6-(pyridoxal phosphate)lysine (0.96) | 0.0 | 5.0 | 0.0 | 6438.0 | 0.15 | 6443.0 |
| N6-carboxylysine (0.88) | 0.0 | 2.0 | 0.0 | 1598.0 | 0.06 | 1600.0 |
| 4-aspartylphosphate (0.85) | 0.0 | 3.0 | 0.0 | 924.0 | 0.05 | 927.0 |
| 3-methylthioaspartic acid (1) | 1.0 | 0.0 | 0.0 | 742.0 | 0.13 | 743.0 |
| Phosphohistidine (0.65) | 0.0 | 1.0 | 0.0 | 600.0 | 0.31 | 601.0 |
| Nitrated tyrosine (0.69) | 1.0 | 0.0 | 0.0 | 324.0 | 0.25 | 325.0 |
| S-nitrosocysteine (0.8) | 3.0 | 0.0 | 0.0 | 264.0 | 0.17 | 267.0 |
| N6-(retinylidene)lysine (1) | 0.0 | 0.0 | 0.0 | 245.0 | 0.06 | 245.0 |
| Methionine sulfoxide (0.99) | 0.0 | 8.0 | 0.0 | 167.0 | 0.39 | 175.0 |
| N6-(3,6-diaminohexanoyl)-5-hydroxylysine (1) | 0.0 | 0.0 | 0.0 | 152.0 | 0.23 | 152.0 |
| 5-glutamyl glycerylphosphorylethanolamine (1) | 0.0 | 5.0 | 0.0 | 119.0 | 0.32 | 124.0 |
| Glycine radical (0.93) | 0.0 | 0.0 | 0.0 | 123.0 | 0.0 | 123.0 |
| Cysteine sulfenic acid (-SOH) (1) | 0.0 | 0.0 | 0.0 | 112.0 | 0.23 | 112.0 |
| N4-methylasparagine (0.96) | 0.0 | 0.0 | 0.0 | 104.0 | 0.33 | 104.0 |Supplementary Figure 12

## Slide 13
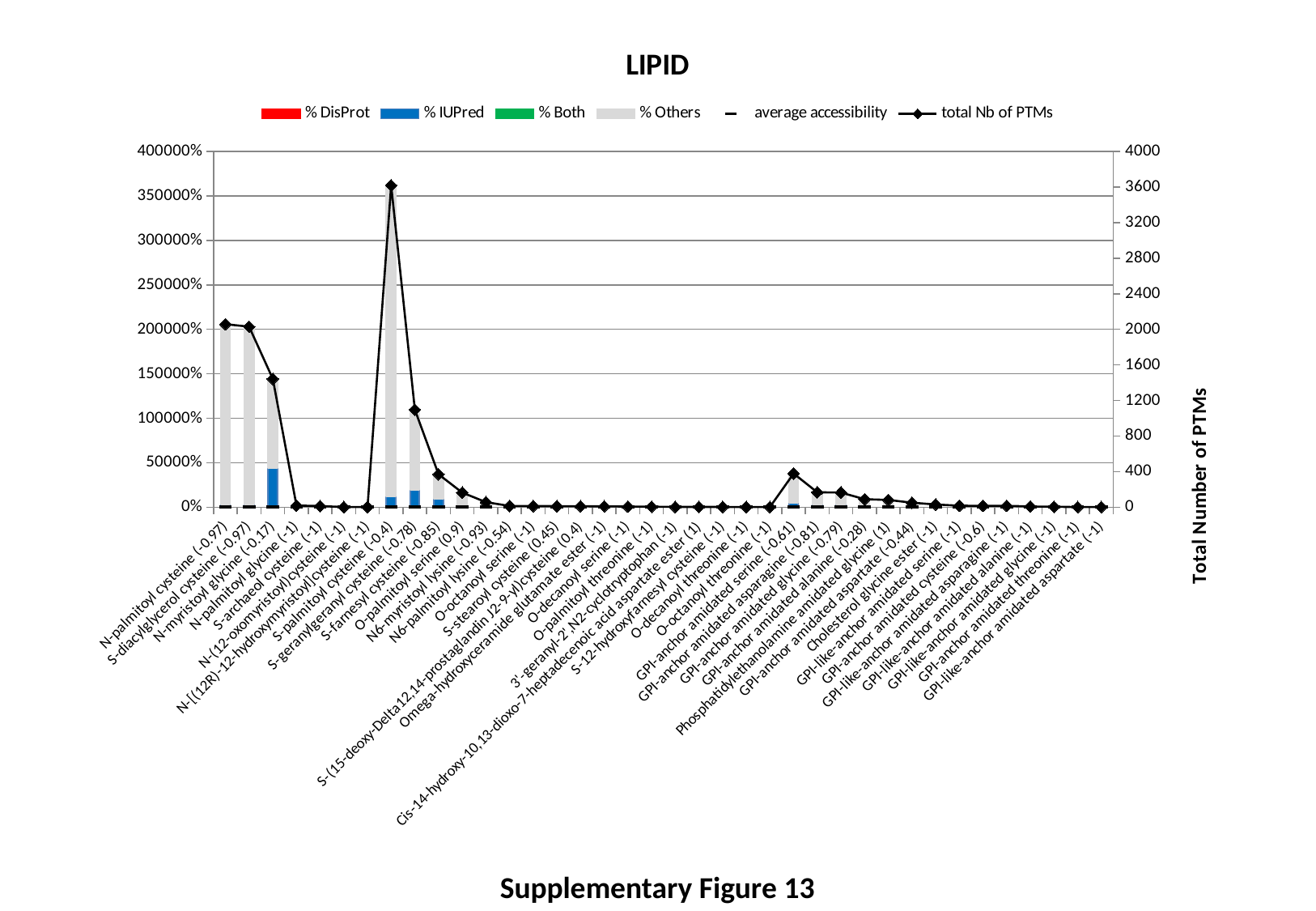

### Chart: LIPID
| Category | % DisProt | % IUPred | % Both | % Others | average accessibility | total Nb of PTMs |
|---|---|---|---|---|---|---|
| N-palmitoyl cysteine (-0.97) | 2.0 | 29.0 | 0.0 | 2027.0 | 0.82 | 2058.0 |
| S-diacylglycerol cysteine (-0.97) | 2.0 | 29.0 | 0.0 | 1999.0 | 0.82 | 2030.0 |
| N-myristoyl glycine (-0.17) | 7.0 | 429.0 | 2.0 | 1005.0 | 1.07 | 1443.0 |
| N-palmitoyl glycine (-1) | 0.0 | 16.0 | 0.0 | 2.0 | 0.0 | 18.0 |
| S-archaeol cysteine (-1) | 0.0 | 2.0 | 0.0 | 10.0 | 0.0 | 12.0 |
| N-(12-oxomyristoyl)cysteine (-1) | 0.0 | 0.0 | 0.0 | 1.0 | 0.0 | 1.0 |
| N-[(12R)-12-hydroxymyristoyl]cysteine (-1) | 0.0 | 0.0 | 0.0 | 1.0 | 0.0 | 1.0 |
| S-palmitoyl cysteine (-0.4) | 10.0 | 109.0 | 0.0 | 3500.0 | 0.53 | 3619.0 |
| S-geranylgeranyl cysteine (-0.78) | 0.0 | 197.0 | 0.0 | 899.0 | 0.62 | 1096.0 |
| S-farnesyl cysteine (-0.85) | 4.0 | 90.0 | 0.0 | 275.0 | 0.16 | 369.0 |
| O-palmitoyl serine (0.9) | 0.0 | 0.0 | 0.0 | 164.0 | 0.16 | 164.0 |
| N6-myristoyl lysine (-0.93) | 0.0 | 6.0 | 0.0 | 50.0 | 0.0 | 56.0 |
| N6-palmitoyl lysine (-0.54) | 0.0 | 0.0 | 0.0 | 13.0 | 0.59 | 13.0 |
| O-octanoyl serine (-1) | 0.0 | 0.0 | 0.0 | 12.0 | 0.0 | 12.0 |
| S-stearoyl cysteine (0.45) | 0.0 | 0.0 | 0.0 | 11.0 | 0.42 | 11.0 |
| S-(15-deoxy-Delta12,14-prostaglandin J2-9-yl)cysteine (0.4) | 1.0 | 0.0 | 0.0 | 9.0 | 0.36 | 10.0 |
| Omega-hydroxyceramide glutamate ester (-1) | 0.0 | 5.0 | 0.0 | 4.0 | 0.0 | 9.0 |
| O-decanoyl serine (-1) | 0.0 | 0.0 | 0.0 | 6.0 | 0.0 | 6.0 |
| O-palmitoyl threonine (-1) | 0.0 | 0.0 | 0.0 | 5.0 | 0.0 | 5.0 |
| 3'-geranyl-2',N2-cyclotryptophan (-1) | 0.0 | 0.0 | 0.0 | 2.0 | 0.0 | 2.0 |
| Cis-14-hydroxy-10,13-dioxo-7-heptadecenoic acid aspartate ester (1) | 0.0 | 0.0 | 0.0 | 2.0 | 0.42 | 2.0 |
| S-12-hydroxyfarnesyl cysteine (-1) | 0.0 | 2.0 | 0.0 | 0.0 | 0.0 | 2.0 |
| O-decanoyl threonine (-1) | 0.0 | 0.0 | 0.0 | 1.0 | 0.0 | 1.0 |
| O-octanoyl threonine (-1) | 0.0 | 0.0 | 0.0 | 1.0 | 0.0 | 1.0 |
| GPI-anchor amidated serine (-0.61) | 0.0 | 50.0 | 0.0 | 328.0 | 0.63 | 378.0 |
| GPI-anchor amidated asparagine (-0.81) | 0.0 | 31.0 | 0.0 | 136.0 | 0.73 | 167.0 |
| GPI-anchor amidated glycine (-0.79) | 0.0 | 28.0 | 0.0 | 136.0 | 1.01 | 164.0 |
| GPI-anchor amidated alanine (-0.28) | 0.0 | 7.0 | 0.0 | 82.0 | 0.66 | 89.0 |
| Phosphatidylethanolamine amidated glycine (1) | 0.0 | 0.0 | 0.0 | 81.0 | 0.86 | 81.0 |
| GPI-anchor amidated aspartate (-0.44) | 0.0 | 7.0 | 0.0 | 43.0 | 0.78 | 50.0 |
| Cholesterol glycine ester (-1) | 0.0 | 0.0 | 0.0 | 30.0 | 0.0 | 30.0 |
| GPI-like-anchor amidated serine (-1) | 0.0 | 6.0 | 0.0 | 10.0 | 0.0 | 16.0 |
| GPI-anchor amidated cysteine (-0.6) | 0.0 | 3.0 | 0.0 | 12.0 | 0.13 | 15.0 |
| GPI-like-anchor amidated asparagine (-1) | 0.0 | 4.0 | 0.0 | 11.0 | 0.0 | 15.0 |
| GPI-like-anchor amidated alanine (-1) | 0.0 | 2.0 | 0.0 | 5.0 | 0.0 | 7.0 |
| GPI-like-anchor amidated glycine (-1) | 0.0 | 3.0 | 0.0 | 1.0 | 0.0 | 4.0 |
| GPI-anchor amidated threonine (-1) | 0.0 | 0.0 | 0.0 | 1.0 | 0.0 | 1.0 |
| GPI-like-anchor amidated aspartate (-1) | 0.0 | 0.0 | 0.0 | 1.0 | 0.0 | 1.0 |Supplementary Figure 13
