# Supplementary material for: Single‐residue posttranslational modification sites at the N‐terminus, C‐terminus or in‐between: To be or not to be exposed for enzyme access
Source: Proteomics. 2015 Jul 14;15(14):2525–46. doi: 10.1002/pmic.201400633 (PMC4745020; doi:10.1002/pmic.201400633)
Supplement: Supplementary file 2 — Table 1. Summary of PTM analysis annotated under MOD_RES with a negative score and low accessibility. Table 2. Summary of PTM analysis annotated under MOD_RES mostly with a positive score and low accessibility. Table 3. Summary of lipid PTM sites hitting DisProt‐ or IUpred‐predicted regions [file PMIC-15-2525-s002.docx]

**Supplementary Material**

**Supplementary Table 1:** Summary of PTM analysis annotated under MOD_RES with a negative score and low accessibility.

|  | **Total Nb of Annotated PTM Sites** | **Nb of Annotated PTM Sites Matching Structure** | **Score** | **Accessibility** | **PDB:chain (hits)** |
| --- | --- | --- | --- | --- | --- |
| N-methylleucine | 3 | 1 | -0.333 | 0.37 | 3QT4:A |
| N-methylproline | 11 | 1 | -0.818 | 0.34 | 4JHP:C |
| N-acetylproline | 315 | 8 | -0.949 | 0.13 | 3KER:A\|3KAN:A |

**Supplementary Table 2:** Summary of PTM analysis annotated under MOD_RES mostly with a positive score and low accessibility.

**Supplementary Table 3:** Summary of lipid PTM sites hitting DisProt- or IUpred-predicted regions

| **PTM (LIPID)** | **DisProt** | **IUPred** | **Both** | **Others** | **nb. of instances** | **Localization** |
| --- | --- | --- | --- | --- | --- | --- |
| N-palmitoyl cysteine | 2 (0.1%) | 29 (1.4%) | 0 (0.0%) | 2027 (98.5%) | 2058 | N-terminal |
| S-diacylglycerol cysteine | 2 (0.1%) | 29 (1.4%) | 0 (0.0%) | 1999 (98.5%) | 2030 | N-terminal |
| N-myristoyl glycine | 7 (0.5%) | 429 (29.7%) | 2 (0.1%) | 1005 (69.6%) | 1443 | N-terminal |
| N-palmitoyl glycine | 0 (0.0%) | 16 (88.9%) | 0 (0.0%) | 2 (11.1%) | 18 | N-terminal |
| S-archaeol cysteine | 0 (0.0%) | 2 (16.7%) | 0 (0.0%) | 10 (83.3%) | 12 | N-terminal |
| N-(12-oxomyristoyl)cysteine | 0 (0.0%) | 0 (0.0%) | 0 (0.0%) | 1 (100.0%) | 1 | N-terminal |
| N-[(12R)-12-hydroxymyristoyl]cysteine | 0 (0.0%) | 0 (0.0%) | 0 (0.0%) | 1 (100.0%) | 1 | N-terminal |
| S-palmitoyl cysteine | 10 (0.3%) | 109 (3.0%) | 0 (0.0%) | 3500 (96.7%) | 3619 | Anywhere |
| S-geranylgeranyl cysteine | 0 (0.0%) | 197 (18.0%) | 0 (0.0%) | 899 (82.0%) | 1096 | Anywhere |
| S-farnesyl cysteine | 4 (1.1%) | 90 (24.4%) | 0 (0.0%) | 275 (74.5%) | 369 | Anywhere |
| O-palmitoyl serine | 0 (0.0%) | 0 (0.0%) | 0 (0.0%) | 164 (100.0%) | 164 | Anywhere |
| N6-myristoyl lysine | 0 (0.0%) | 6 (10.7%) | 0 (0.0%) | 50 (89.3%) | 56 | Anywhere |
| N6-palmitoyl lysine | 0 (0.0%) | 0 (0.0%) | 0 (0.0%) | 13 (100.0%) | 13 | Anywhere |
| O-octanoyl serine | 0 (0.0%) | 0 (0.0%) | 0 (0.0%) | 12 (100.0%) | 12 | Anywhere |
| S-stearoyl cysteine | 0 (0.0%) | 0 (0.0%) | 0 (0.0%) | 11 (100.0%) | 11 | Anywhere |
| S-(15-deoxy-Delta12,14-prostaglandin J2-9-yl)cysteine | 1 (10.0%) | 0 (0.0%) | 0 (0.0%) | 9 (90.0%) | 10 | Anywhere |
| Omega-hydroxyceramide glutamate ester | 0 (0.0%) | 5 (55.6%) | 0 (0.0%) | 4 (44.4%) | 9 | Anywhere |
| O-decanoyl serine | 0 (0.0%) | 0 (0.0%) | 0 (0.0%) | 6 (100.0%) | 6 | Anywhere |
| O-palmitoyl threonine | 0 (0.0%) | 0 (0.0%) | 0 (0.0%) | 5 (100.0%) | 5 | Anywhere |
| 3'-geranyl-2',N2-cyclotryptophan | 0 (0.0%) | 0 (0.0%) | 0 (0.0%) | 2 (100.0%) | 2 | Anywhere |
| Cis-14-hydroxy-10,13-dioxo-7-heptadecenoic acid aspartate ester | 0 (0.0%) | 0 (0.0%) | 0 (0.0%) | 2 (100.0%) | 2 | Anywhere |
| S-12-hydroxyfarnesyl cysteine | 0 (0.0%) | 2 (100.0%) | 0 (0.0%) | 0 (0.0%) | 2 | Anywhere |
| O-decanoyl threonine | 0 (0.0%) | 0 (0.0%) | 0 (0.0%) | 1 (100.0%) | 1 | Anywhere |
| O-octanoyl threonine | 0 (0.0%) | 0 (0.0%) | 0 (0.0%) | 1 (100.0%) | 1 | Anywhere |
| GPI-anchor amidated serine | 0 (0.0%) | 50 (13.2%) | 0 (0.0%) | 328 (86.8%) | 378 | C-terminal |
| GPI-anchor amidated asparagine | 0 (0.0%) | 31 (18.6%) | 0 (0.0%) | 136 (81.4%) | 167 | C-terminal |
| GPI-anchor amidated glycine | 0 (0.0%) | 28 (17.1%) | 0 (0.0%) | 136 (82.9%) | 164 | C-terminal |
| GPI-anchor amidated alanine | 0 (0.0%) | 7 (7.9%) | 0 (0.0%) | 82 (92.1%) | 89 | C-terminal |
| Phosphatidylethanolamine amidated glycine | 0 (0.0%) | 0 (0.0%) | 0 (0.0%) | 81 (100.0%) | 81 | C-terminal |
| GPI-anchor amidated aspartate | 0 (0.0%) | 7 (14.0%) | 0 (0.0%) | 43 (86.0%) | 50 | C-terminal |
| Cholesterol glycine ester | 0 (0.0%) | 0 (0.0%) | 0 (0.0%) | 30 (100.0%) | 30 | C-terminal |
| GPI-like-anchor amidated serine | 0 (0.0%) | 6 (37.5%) | 0 (0.0%) | 10 (62.5%) | 16 | C-terminal |
| GPI-anchor amidated cysteine | 0 (0.0%) | 3 (20.0%) | 0 (0.0%) | 12 (80.0%) | 15 | C-terminal |
| GPI-like-anchor amidated asparagine | 0 (0.0%) | 4 (26.7%) | 0 (0.0%) | 11 (73.3%) | 15 | C-terminal |
| GPI-like-anchor amidated alanine | 0 (0.0%) | 2 (28.6%) | 0 (0.0%) | 5 (71.4%) | 7 | C-terminal |
| GPI-like-anchor amidated glycine | 0 (0.0%) | 3 (75.0%) | 0 (0.0%) | 1 (25.0%) | 4 | C-terminal |
| GPI-anchor amidated threonine | 0 (0.0%) | 0 (0.0%) | 0 (0.0%) | 1 (100.0%) | 1 | C-terminal |
| GPI-like-anchor amidated aspartate | 0 (0.0%) | 0 (0.0%) | 0 (0.0%) | 1 (100.0%) | 1 | C-terminal |

**Supplementary Table 4:** Summary of MOD_RES PTM sites hitting DisProt- or IUpred-predicted regions

| **PTM (MOD_RES)** | **DisProt** | **IUPred** | **Both** | **Others** | **Nb. of instances** | **Localization** |
| --- | --- | --- | --- | --- | --- | --- |
| Phosphoserine | 132 (0.2%) | 46365 (62.6%) | 488 (0.7%) | 27084 (36.6%) | 74069 | Anywhere |
| Phosphothreonine | 35 (0.2%) | 8907 (51.9%) | 100 (0.6%) | 8130 (47.3%) | 17172 | Anywhere |
| N6-acetyllysine | 19 (0.1%) | 3767 (25.2%) | 31 (0.2%) | 11161 (74.5%) | 14978 | Anywhere |
| Phosphotyrosine | 31 (0.3%) | 1795 (20.0%) | 21 (0.2%) | 7123 (79.4%) | 8970 | Anywhere |
| N6-(pyridoxal phosphate)lysine | 0 (0.0%) | 5 (0.1%) | 0 (0.0%) | 6438 (99.9%) | 6443 | Anywhere |
| 4-hydroxyproline | 0 (0.0%) | 1073 (59.4%) | 1 (0.1%) | 732 (40.5%) | 1806 | Anywhere |
| N6-carboxylysine | 0 (0.0%) | 2 (0.1%) | 0 (0.0%) | 1598 (99.9%) | 1600 | Anywhere |
| N5-methylglutamine | 0 (0.0%) | 651 (50.2%) | 0 (0.0%) | 647 (49.8%) | 1298 | Anywhere |
| N6,N6,N6-trimethyllysine | 0 (0.0%) | 319 (25.2%) | 0 (0.0%) | 948 (74.8%) | 1267 | Anywhere |
| 4-carboxyglutamate | 2 (0.2%) | 125 (10.0%) | 1 (0.1%) | 1126 (89.8%) | 1254 | Anywhere |
| N6-methyllysine | 0 (0.0%) | 631 (59.0%) | 1 (0.1%) | 438 (40.9%) | 1070 | Anywhere |
| Sulfotyrosine | 2 (0.2%) | 180 (17.7%) | 8 (0.8%) | 829 (81.4%) | 1019 | Anywhere |
| O-(pantetheine 4'-phosphoryl)serine | 0 (0.0%) | 1 (0.1%) | 0 (0.0%) | 1011 (99.9%) | 1012 | Anywhere |
| 4-aspartylphosphate | 0 (0.0%) | 3 (0.3%) | 0 (0.0%) | 924 (99.7%) | 927 | Anywhere |
| Asymmetric dimethylarginine | 1 (0.1%) | 652 (81.6%) | 7 (0.9%) | 139 (17.4%) | 799 | Anywhere |
| N6,N6-dimethyllysine | 0 (0.0%) | 435 (58.0%) | 0 (0.0%) | 315 (42.0%) | 750 | Anywhere |
| 3-methylthioaspartic acid | 1 (0.1%) | 0 (0.0%) | 0 (0.0%) | 742 (99.9%) | 743 | Anywhere |
| Phosphohistidine | 0 (0.0%) | 1 (0.2%) | 0 (0.0%) | 600 (99.8%) | 601 | Anywhere |
| N6-lipoyllysine | 0 (0.0%) | 62 (10.6%) | 0 (0.0%) | 523 (89.4%) | 585 | Anywhere |
| N6-crotonyl-L-lysine | 0 (0.0%) | 417 (76.0%) | 0 (0.0%) | 132 (24.0%) | 549 | Anywhere |
| 2-(S-cysteinyl)pyruvic acid O-phosphothioketal | 0 (0.0%) | 0 (0.0%) | 0 (0.0%) | 530 (100.0%) | 530 | Anywhere |
| S-(dipyrrolylmethanemethyl)cysteine | 0 (0.0%) | 0 (0.0%) | 0 (0.0%) | 471 (100.0%) | 471 | Anywhere |
| N6-methylated lysine | 0 (0.0%) | 291 (77.6%) | 0 (0.0%) | 84 (22.4%) | 375 | Anywhere |
| Omega-N-methylated arginine | 0 (0.0%) | 193 (56.3%) | 2 (0.6%) | 148 (43.1%) | 343 | Anywhere |
| Nitrated tyrosine | 1 (0.3%) | 0 (0.0%) | 0 (0.0%) | 324 (99.7%) | 325 | Anywhere |
| Hydroxyproline | 0 (0.0%) | 223 (70.1%) | 1 (0.3%) | 94 (29.6%) | 318 | Anywhere |
| 5-hydroxylysine | 0 (0.0%) | 267 (95.4%) | 0 (0.0%) | 13 (4.6%) | 280 | Anywhere |
| S-nitrosocysteine | 3 (1.1%) | 0 (0.0%) | 0 (0.0%) | 264 (98.9%) | 267 | Anywhere |
| Omega-N-methylarginine | 0 (0.0%) | 176 (67.2%) | 7 (2.7%) | 79 (30.2%) | 262 | Anywhere |
| N6-(retinylidene)lysine | 0 (0.0%) | 0 (0.0%) | 0 (0.0%) | 245 (100.0%) | 245 | Anywhere |
| Deamidated asparagine | 0 (0.0%) | 20 (8.6%) | 0 (0.0%) | 212 (91.4%) | 232 | Anywhere |
| Deamidated glutamine | 4 (2.0%) | 19 (9.4%) | 5 (2.5%) | 174 (86.1%) | 202 | Anywhere |
| N6-malonyllysine | 0 (0.0%) | 20 (10.0%) | 0 (0.0%) | 180 (90.0%) | 200 | Anywhere |
| Pentaglycyl murein peptidoglycan amidated threonine | 0 (0.0%) | 155 (77.9%) | 0 (0.0%) | 44 (22.1%) | 199 | Anywhere |
| Citrulline | 3 (1.6%) | 117 (64.3%) | 21 (11.5%) | 41 (22.5%) | 182 | Anywhere |
| Methionine sulfoxide | 0 (0.0%) | 8 (4.6%) | 0 (0.0%) | 167 (95.4%) | 175 | Anywhere |
| N6-succinyllysine | 0 (0.0%) | 4 (2.5%) | 0 (0.0%) | 154 (97.5%) | 158 | Anywhere |
| ADP-ribosylarginine | 0 (0.0%) | 0 (0.0%) | 0 (0.0%) | 155 (100.0%) | 155 | Anywhere |
| N6-(3,6-diaminohexanoyl)-5-hydroxylysine | 0 (0.0%) | 0 (0.0%) | 0 (0.0%) | 152 (100.0%) | 152 | Anywhere |
| O-(5'-phospho-RNA)-tyrosine | 0 (0.0%) | 22 (14.6%) | 0 (0.0%) | 129 (85.4%) | 151 | Anywhere |
| Allysine | 0 (0.0%) | 59 (41.0%) | 0 (0.0%) | 85 (59.0%) | 144 | Anywhere |
| Symmetric dimethylarginine | 0 (0.0%) | 106 (75.7%) | 5 (3.6%) | 29 (20.7%) | 140 | Anywhere |
| 3',4'-dihydroxyphenylalanine | 0 (0.0%) | 0 (0.0%) | 0 (0.0%) | 130 (100.0%) | 130 | Anywhere |
| 5-glutamyl glycerylphosphorylethanolamine | 0 (0.0%) | 5 (4.0%) | 0 (0.0%) | 119 (96.0%) | 124 | Anywhere |
| Glycine radical | 0 (0.0%) | 0 (0.0%) | 0 (0.0%) | 123 (100.0%) | 123 | Anywhere |
| Dimethylated arginine | 0 (0.0%) | 88 (73.3%) | 1 (0.8%) | 31 (25.8%) | 120 | Anywhere |
| Cysteine sulfenic acid (-SOH) | 0 (0.0%) | 0 (0.0%) | 0 (0.0%) | 112 (100.0%) | 112 | Anywhere |
| O-(phosphoribosyl dephospho-coenzyme A)serine | 0 (0.0%) | 1 (0.9%) | 0 (0.0%) | 111 (99.1%) | 112 | Anywhere |
| Hypusine | 0 (0.0%) | 4 (3.7%) | 0 (0.0%) | 104 (96.3%) | 108 | Anywhere |
| PolyADP-ribosyl glutamic acid | 0 (0.0%) | 26 (24.3%) | 0 (0.0%) | 81 (75.7%) | 107 | Anywhere |
| Aspartyl aldehyde | 0 (0.0%) | 0 (0.0%) | 0 (0.0%) | 104 (100.0%) | 104 | Anywhere |
| N4-methylasparagine | 0 (0.0%) | 0 (0.0%) | 0 (0.0%) | 104 (100.0%) | 104 | Anywhere |
| 7'-hydroxytryptophan | 0 (0.0%) | 64 (68.1%) | 0 (0.0%) | 30 (31.9%) | 94 | Anywhere |
| Diphthamide | 0 (0.0%) | 5 (5.4%) | 0 (0.0%) | 87 (94.6%) | 92 | Anywhere |
| 6'-bromotryptophan | 0 (0.0%) | 0 (0.0%) | 0 (0.0%) | 89 (100.0%) | 89 | Anywhere |
| N6-biotinyllysine | 0 (0.0%) | 2 (2.4%) | 0 (0.0%) | 80 (97.6%) | 82 | Anywhere |
| S-8alpha-FAD cysteine | 0 (0.0%) | 0 (0.0%) | 0 (0.0%) | 81 (100.0%) | 81 | Anywhere |
| Tele-methylhistidine | 0 (0.0%) | 0 (0.0%) | 0 (0.0%) | 79 (100.0%) | 79 | Anywhere |
| (3S)-3-hydroxyasparagine | 1 (1.4%) | 6 (8.2%) | 0 (0.0%) | 66 (90.4%) | 73 | Anywhere |
| O-AMP-tyrosine | 1 (1.5%) | 17 (25.4%) | 0 (0.0%) | 49 (73.1%) | 67 | Anywhere |
| 3-oxoalanine (Cys) | 0 (0.0%) | 0 (0.0%) | 0 (0.0%) | 63 (100.0%) | 63 | Anywhere |
| Glutamate methyl ester (Glu) | 0 (0.0%) | 23 (37.1%) | 0 (0.0%) | 39 (62.9%) | 62 | Anywhere |
| S-glutathionyl cysteine | 0 (0.0%) | 0 (0.0%) | 0 (0.0%) | 59 (100.0%) | 59 | Anywhere |
| Tele-8alpha-FAD histidine | 0 (0.0%) | 1 (1.7%) | 0 (0.0%) | 58 (98.3%) | 59 | Anywhere |
| 3-hydroxyproline | 0 (0.0%) | 56 (96.6%) | 0 (0.0%) | 2 (3.4%) | 58 | Anywhere |
| (3R,4S)-3,4-dihydroxyproline | 0 (0.0%) | 0 (0.0%) | 0 (0.0%) | 55 (100.0%) | 55 | Anywhere |
| FMN phosphoryl threonine | 0 (0.0%) | 0 (0.0%) | 0 (0.0%) | 54 (100.0%) | 54 | Anywhere |
| 2',4',5'-topaquinone | 0 (0.0%) | 0 (0.0%) | 0 (0.0%) | 53 (100.0%) | 53 | Anywhere |
| (3R)-3-hydroxyaspartate | 0 (0.0%) | 0 (0.0%) | 0 (0.0%) | 48 (100.0%) | 48 | Anywhere |
| Pros-8alpha-FAD histidine | 0 (0.0%) | 4 (9.5%) | 0 (0.0%) | 38 (90.5%) | 42 | Anywhere |
| (3R)-3-hydroxyasparagine | 0 (0.0%) | 0 (0.0%) | 0 (0.0%) | 38 (100.0%) | 38 | Anywhere |
| O-UMP-tyrosine | 0 (0.0%) | 0 (0.0%) | 0 (0.0%) | 37 (100.0%) | 37 | Anywhere |
| S-4a-FMN cysteine | 0 (0.0%) | 0 (0.0%) | 0 (0.0%) | 35 (100.0%) | 35 | Anywhere |
| ADP-ribosylcysteine | 0 (0.0%) | 0 (0.0%) | 0 (0.0%) | 33 (100.0%) | 33 | Anywhere |
| Glutamate methyl ester (Gln) | 0 (0.0%) | 16 (51.6%) | 0 (0.0%) | 15 (48.4%) | 31 | Anywhere |
| Cysteine persulfide | 1 (4.3%) | 0 (0.0%) | 0 (0.0%) | 22 (95.7%) | 23 | Anywhere |
| Cysteine sulfinic acid (-SO2H) | 0 (0.0%) | 0 (0.0%) | 0 (0.0%) | 19 (100.0%) | 19 | Anywhere |
| N6-murein peptidoglycan lysine | 0 (0.0%) | 5 (26.3%) | 0 (0.0%) | 14 (73.7%) | 19 | Anywhere |
| Pros-methylhistidine | 0 (0.0%) | 5 (26.3%) | 0 (0.0%) | 14 (73.7%) | 19 | Anywhere |
| S-methylcysteine | 0 (0.0%) | 0 (0.0%) | 0 (0.0%) | 19 (100.0%) | 19 | Anywhere |
| Methylhistidine | 0 (0.0%) | 12 (66.7%) | 0 (0.0%) | 6 (33.3%) | 18 | Anywhere |
| (3S)-3-hydroxyhistidine | 0 (0.0%) | 13 (76.5%) | 0 (0.0%) | 4 (23.5%) | 17 | Anywhere |
| O-(2-cholinephosphoryl)serine | 0 (0.0%) | 1 (6.7%) | 0 (0.0%) | 14 (93.3%) | 15 | Anywhere |
| N6-poly(methylaminopropyl)lysine | 0 (0.0%) | 11 (84.6%) | 0 (0.0%) | 2 (15.4%) | 13 | Anywhere |
| O-(5'-phospho-DNA)-serine | 0 (0.0%) | 0 (0.0%) | 0 (0.0%) | 13 (100.0%) | 13 | Anywhere |
| Thyroxine | 0 (0.0%) | 0 (0.0%) | 0 (0.0%) | 13 (100.0%) | 13 | Anywhere |
| 5-methylarginine | 0 (0.0%) | 0 (0.0%) | 0 (0.0%) | 12 (100.0%) | 12 | Anywhere |
| O-(5'-phospho-RNA)-serine | 0 (0.0%) | 0 (0.0%) | 0 (0.0%) | 9 (100.0%) | 9 | Anywhere |
| S-cysteinyl cysteine | 0 (0.0%) | 0 (0.0%) | 0 (0.0%) | 9 (100.0%) | 9 | Anywhere |
| Tryptophylquinone | 0 (0.0%) | 0 (0.0%) | 0 (0.0%) | 9 (100.0%) | 9 | Anywhere |
| 4,5-dihydroxylysine | 0 (0.0%) | 0 (0.0%) | 0 (0.0%) | 8 (100.0%) | 8 | Anywhere |
| 5-glutamyl polyglycine | 0 (0.0%) | 8 (100.0%) | 0 (0.0%) | 0 (0.0%) | 8 | Anywhere |
| ADP-ribosylasparagine | 0 (0.0%) | 0 (0.0%) | 0 (0.0%) | 8 (100.0%) | 8 | Anywhere |
| Cysteine methyl disulfide | 0 (0.0%) | 0 (0.0%) | 0 (0.0%) | 8 (100.0%) | 8 | Anywhere |
| ADP-ribosylserine | 0 (0.0%) | 0 (0.0%) | 0 (0.0%) | 7 (100.0%) | 7 | Anywhere |
| N6-formyllysine | 0 (0.0%) | 0 (0.0%) | 0 (0.0%) | 6 (100.0%) | 6 | Anywhere |
| 3-hydroxytryptophan | 0 (0.0%) | 0 (0.0%) | 0 (0.0%) | 5 (100.0%) | 5 | Anywhere |
| N5-methylarginine | 0 (0.0%) | 0 (0.0%) | 0 (0.0%) | 5 (100.0%) | 5 | Anywhere |
| N6,N6,N6-trimethyl-5-hydroxylysine | 0 (0.0%) | 5 (100.0%) | 0 (0.0%) | 0 (0.0%) | 5 | Anywhere |
| S-(4-hydroxycinnamyl)cysteine | 0 (0.0%) | 0 (0.0%) | 0 (0.0%) | 5 (100.0%) | 5 | Anywhere |
| 3-oxoalanine (Ser) | 0 (0.0%) | 0 (0.0%) | 0 (0.0%) | 4 (100.0%) | 4 | Anywhere |
| N6-1-carboxyethyl lysine | 0 (0.0%) | 4 (100.0%) | 0 (0.0%) | 0 (0.0%) | 4 | Anywhere |
| Phosphoarginine | 0 (0.0%) | 1 (25.0%) | 0 (0.0%) | 3 (75.0%) | 4 | Anywhere |
| S-selanylcysteine | 0 (0.0%) | 0 (0.0%) | 0 (0.0%) | 4 (100.0%) | 4 | Anywhere |
| Triiodothyronine | 0 (0.0%) | 2 (50.0%) | 0 (0.0%) | 2 (50.0%) | 4 | Anywhere |
| O-AMP-threonine | 1 (33.3%) | 0 (0.0%) | 0 (0.0%) | 2 (66.7%) | 3 | Anywhere |
| 3-hydroxyvaline | 0 (0.0%) | 0 (0.0%) | 0 (0.0%) | 3 (100.0%) | 3 | Anywhere |
| O-(sn-1-glycerophosphoryl)serine | 0 (0.0%) | 0 (0.0%) | 0 (0.0%) | 3 (100.0%) | 3 | Anywhere |
| O-acetylserine | 0 (0.0%) | 0 (0.0%) | 0 (0.0%) | 3 (100.0%) | 3 | Anywhere |
| O-acetylthreonine | 0 (0.0%) | 0 (0.0%) | 0 (0.0%) | 3 (100.0%) | 3 | Anywhere |
| O-methylthreonine | 0 (0.0%) | 0 (0.0%) | 0 (0.0%) | 3 (100.0%) | 3 | Anywhere |
| Phosphocysteine | 0 (0.0%) | 0 (0.0%) | 0 (0.0%) | 3 (100.0%) | 3 | Anywhere |
| Sulfoserine | 0 (0.0%) | 0 (0.0%) | 0 (0.0%) | 3 (100.0%) | 3 | Anywhere |
| (3R,4R)-4,5-dihydroxyisoleucine | 0 (0.0%) | 0 (0.0%) | 0 (0.0%) | 2 (100.0%) | 2 | Anywhere |
| (3R,4S)-4-hydroxyisoleucine | 0 (0.0%) | 0 (0.0%) | 0 (0.0%) | 2 (100.0%) | 2 | Anywhere |
| (3S)-3-hydroxyaspartate | 0 (0.0%) | 0 (0.0%) | 0 (0.0%) | 2 (100.0%) | 2 | Anywhere |
| 3,4-dihydroxyarginine | 0 (0.0%) | 0 (0.0%) | 0 (0.0%) | 2 (100.0%) | 2 | Anywhere |
| FMN phosphoryl serine | 0 (0.0%) | 0 (0.0%) | 0 (0.0%) | 2 (100.0%) | 2 | Anywhere |
| N6-(ADP-ribosyl)lysine | 0 (0.0%) | 2 (100.0%) | 0 (0.0%) | 0 (0.0%) | 2 | Anywhere |
| S-6-FMN cysteine | 0 (0.0%) | 0 (0.0%) | 0 (0.0%) | 2 (100.0%) | 2 | Anywhere |
| (3R)-3-hydroxyarginine | 0 (0.0%) | 0 (0.0%) | 0 (0.0%) | 1 (100.0%) | 1 | Anywhere |
| (3R,4R)-3,4-dihydroxyproline | 0 (0.0%) | 0 (0.0%) | 0 (0.0%) | 1 (100.0%) | 1 | Anywhere |
| (3S,4R)-3,4-dihydroxyisoleucine | 0 (0.0%) | 0 (0.0%) | 0 (0.0%) | 1 (100.0%) | 1 | Anywhere |
| (4R)-4,5-dihydroxyleucine | 0 (0.0%) | 0 (0.0%) | 0 (0.0%) | 1 (100.0%) | 1 | Anywhere |
| 2'-methylsulfonyltryptophan | 0 (0.0%) | 0 (0.0%) | 0 (0.0%) | 1 (100.0%) | 1 | Anywhere |
| 3,4-dihydroxyproline | 0 (0.0%) | 0 (0.0%) | 0 (0.0%) | 1 (100.0%) | 1 | Anywhere |
| 3-hydroxyphenylalanine | 0 (0.0%) | 0 (0.0%) | 0 (0.0%) | 1 (100.0%) | 1 | Anywhere |
| 4,5,5'-trihydroxyleucine | 0 (0.0%) | 0 (0.0%) | 0 (0.0%) | 1 (100.0%) | 1 | Anywhere |
| 4-hydroxyarginine | 0 (0.0%) | 0 (0.0%) | 0 (0.0%) | 1 (100.0%) | 1 | Anywhere |
| 4-hydroxyglutamate | 0 (0.0%) | 0 (0.0%) | 0 (0.0%) | 1 (100.0%) | 1 | Anywhere |
| 5-hydroxy-3-methylproline (Ile) | 0 (0.0%) | 0 (0.0%) | 0 (0.0%) | 1 (100.0%) | 1 | Anywhere |
| 6'-chlorotryptophan | 0 (0.0%) | 0 (0.0%) | 0 (0.0%) | 1 (100.0%) | 1 | Anywhere |
| Aminomalonic acid (Ser) | 0 (0.0%) | 0 (0.0%) | 0 (0.0%) | 1 (100.0%) | 1 | Anywhere |
| Beta-decarboxylated aspartate | 0 (0.0%) | 0 (0.0%) | 0 (0.0%) | 1 (100.0%) | 1 | Anywhere |
| Bromohistidine | 0 (0.0%) | 1 (100.0%) | 0 (0.0%) | 0 (0.0%) | 1 | Anywhere |
| Lysino-D-alanine (Lys) | 0 (0.0%) | 0 (0.0%) | 0 (0.0%) | 1 (100.0%) | 1 | Anywhere |
| Methionine sulfone | 0 (0.0%) | 0 (0.0%) | 0 (0.0%) | 1 (100.0%) | 1 | Anywhere |
| N4,N4-dimethylasparagine | 0 (0.0%) | 1 (100.0%) | 0 (0.0%) | 0 (0.0%) | 1 | Anywhere |
| O-(2-aminoethylphosphoryl)serine | 0 (0.0%) | 1 (100.0%) | 0 (0.0%) | 0 (0.0%) | 1 | Anywhere |
| O-(5'-phospho-DNA)-tyrosine | 0 (0.0%) | 0 (0.0%) | 0 (0.0%) | 1 (100.0%) | 1 | Anywhere |
| O-8alpha-FAD tyrosine | 0 (0.0%) | 0 (0.0%) | 0 (0.0%) | 1 (100.0%) | 1 | Anywhere |
| Pentaglycyl murein peptidoglycan amidated alanine | 0 (0.0%) | 1 (100.0%) | 0 (0.0%) | 0 (0.0%) | 1 | Anywhere |
| S-(coelenterazin-3a-yl)cysteine | 0 (0.0%) | 0 (0.0%) | 0 (0.0%) | 1 (100.0%) | 1 | Anywhere |
| S-bacillithiol cysteine disulfide | 0 (0.0%) | 0 (0.0%) | 0 (0.0%) | 1 (100.0%) | 1 | Anywhere |
| Sulfothreonine | 0 (0.0%) | 0 (0.0%) | 0 (0.0%) | 1 (100.0%) | 1 | Anywhere |
| Tele-(1,2,3-trihydroxypropan-2-yl)histidine | 0 (0.0%) | 0 (0.0%) | 0 (0.0%) | 1 (100.0%) | 1 | Anywhere |
| Tele-8alpha-FMN histidine | 0 (0.0%) | 0 (0.0%) | 0 (0.0%) | 1 (100.0%) | 1 | Anywhere |
| Tele-phosphohistidine | 0 (0.0%) | 0 (0.0%) | 0 (0.0%) | 1 (100.0%) | 1 | Anywhere |
| Phenylalanine amide | 0 (0.0%) | 156 (18.7%) | 0 (0.0%) | 680 (81.3%) | 836 | C-terminal |
| Cysteine methyl ester | 4 (0.5%) | 145 (17.5%) | 0 (0.0%) | 680 (82.0%) | 829 | C-terminal |
| Leucine amide | 0 (0.0%) | 128 (16.8%) | 0 (0.0%) | 632 (83.2%) | 760 | C-terminal |
| Valine amide | 0 (0.0%) | 124 (29.9%) | 0 (0.0%) | 291 (70.1%) | 415 | C-terminal |
| Glycine amide | 0 (0.0%) | 22 (9.3%) | 0 (0.0%) | 214 (90.7%) | 236 | C-terminal |
| Arginine amide | 0 (0.0%) | 31 (15.0%) | 0 (0.0%) | 175 (85.0%) | 206 | C-terminal |
| Cysteine amide | 0 (0.0%) | 0 (0.0%) | 0 (0.0%) | 203 (100.0%) | 203 | C-terminal |
| Tryptophan amide | 0 (0.0%) | 81 (41.3%) | 0 (0.0%) | 115 (58.7%) | 196 | C-terminal |
| Asparagine amide | 0 (0.0%) | 6 (3.6%) | 0 (0.0%) | 161 (96.4%) | 167 | C-terminal |
| Isoleucine amide | 0 (0.0%) | 4 (2.6%) | 0 (0.0%) | 152 (97.4%) | 156 | C-terminal |
| Tyrosine amide | 0 (0.0%) | 15 (10.3%) | 0 (0.0%) | 131 (89.7%) | 146 | C-terminal |
| Proline amide | 0 (0.0%) | 81 (56.6%) | 0 (0.0%) | 62 (43.4%) | 143 | C-terminal |
| Methionine amide | 0 (0.0%) | 29 (21.6%) | 0 (0.0%) | 105 (78.4%) | 134 | C-terminal |
| Lysine amide | 0 (0.0%) | 6 (5.5%) | 0 (0.0%) | 103 (94.5%) | 109 | C-terminal |
| Threonine amide | 0 (0.0%) | 42 (40.0%) | 0 (0.0%) | 63 (60.0%) | 105 | C-terminal |
| Serine amide | 0 (0.0%) | 20 (19.4%) | 0 (0.0%) | 83 (80.6%) | 103 | C-terminal |
| Alanine amide | 0 (0.0%) | 5 (8.9%) | 0 (0.0%) | 51 (91.1%) | 56 | C-terminal |
| Glutamine amide | 0 (0.0%) | 4 (7.5%) | 0 (0.0%) | 49 (92.5%) | 53 | C-terminal |
| Leucine methyl ester | 0 (0.0%) | 0 (0.0%) | 0 (0.0%) | 36 (100.0%) | 36 | C-terminal |
| Glutamic acid 1-amide | 0 (0.0%) | 2 (6.9%) | 0 (0.0%) | 27 (93.1%) | 29 | C-terminal |
| Glycyl adenylate | 0 (0.0%) | 0 (0.0%) | 0 (0.0%) | 29 (100.0%) | 29 | C-terminal |
| Histidine amide | 0 (0.0%) | 1 (4.2%) | 0 (0.0%) | 23 (95.8%) | 24 | C-terminal |
| Aspartate 1-(chondroitin 4-sulfate)-ester | 0 (0.0%) | 0 (0.0%) | 0 (0.0%) | 16 (100.0%) | 16 | C-terminal |
| Aspartic acid 1-amide | 0 (0.0%) | 1 (11.1%) | 0 (0.0%) | 8 (88.9%) | 9 | C-terminal |
| 5-glutamyl 2-aminoadipic acid | 0 (0.0%) | 1 (25.0%) | 0 (0.0%) | 3 (75.0%) | 4 | C-terminal |
| 5-glutamyl N2-lysine | 0 (0.0%) | 1 (25.0%) | 0 (0.0%) | 3 (75.0%) | 4 | C-terminal |
| 5-glutamyl N2-arginine | 0 (0.0%) | 0 (0.0%) | 0 (0.0%) | 3 (100.0%) | 3 | C-terminal |
| 5-glutamyl N2-glutamate | 0 (0.0%) | 0 (0.0%) | 0 (0.0%) | 3 (100.0%) | 3 | C-terminal |
| Decarboxylated threonine | 0 (0.0%) | 0 (0.0%) | 0 (0.0%) | 3 (100.0%) | 3 | C-terminal |
| CysO-cysteine adduct | 0 (0.0%) | 0 (0.0%) | 0 (0.0%) | 2 (100.0%) | 2 | C-terminal |
| 1-amino-2-propanone | 0 (0.0%) | 0 (0.0%) | 0 (0.0%) | 1 (100.0%) | 1 | C-terminal |
| Aspartic acid 1-[(3-aminopropyl)(5'-adenosyl)phosphono]amide | 0 (0.0%) | 1 (100.0%) | 0 (0.0%) | 0 (0.0%) | 1 | C-terminal |
| Cyclo[(prolylserin)-O-yl] cysteinate | 0 (0.0%) | 0 (0.0%) | 0 (0.0%) | 1 (100.0%) | 1 | C-terminal |
| Lysine methyl ester | 0 (0.0%) | 0 (0.0%) | 0 (0.0%) | 1 (100.0%) | 1 | C-terminal |
| Serine microcin E492 siderophore ester | 0 (0.0%) | 1 (100.0%) | 0 (0.0%) | 0 (0.0%) | 1 | C-terminal |
| Blocked amino end (Ala) | 0 (0.0%) | 6 (20.0%) | 0 (0.0%) | 24 (80.0%) | 30 | N.A. |
| Blocked amino end (Met) | 0 (0.0%) | 7 (28.0%) | 0 (0.0%) | 18 (72.0%) | 25 | N.A. |
| Blocked amino end (Ser) | 1 (5.6%) | 4 (22.2%) | 0 (0.0%) | 13 (72.2%) | 18 | N.A. |
| Blocked amino end (Thr) | 1 (6.7%) | 1 (6.7%) | 0 (0.0%) | 13 (86.7%) | 15 | N.A. |
| Blocked amino end (Gln) | 0 (0.0%) | 0 (0.0%) | 0 (0.0%) | 6 (100.0%) | 6 | N.A. |
| Blocked amino end (Gly) | 0 (0.0%) | 0 (0.0%) | 0 (0.0%) | 4 (100.0%) | 4 | N.A. |
| Blocked amino end (Pro) | 0 (0.0%) | 3 (100.0%) | 0 (0.0%) | 0 (0.0%) | 3 | N.A. |
| Cysteine derivative | 0 (0.0%) | 0 (0.0%) | 0 (0.0%) | 3 (100.0%) | 3 | N.A. |
| Lysine derivative | 0 (0.0%) | 2 (66.7%) | 0 (0.0%) | 1 (33.3%) | 3 | N.A. |
| Blocked amino end (Xaa) | 0 (0.0%) | 2 (100.0%) | 0 (0.0%) | 0 (0.0%) | 2 | N.A. |
| Glutamine derivative | 0 (0.0%) | 0 (0.0%) | 0 (0.0%) | 2 (100.0%) | 2 | N.A. |
| Blocked amino end (Arg) | 0 (0.0%) | 0 (0.0%) | 0 (0.0%) | 1 (100.0%) | 1 | N.A. |
| Blocked amino end (Asn) | 0 (0.0%) | 0 (0.0%) | 0 (0.0%) | 1 (100.0%) | 1 | N.A. |
| Blocked amino end (Asp) | 0 (0.0%) | 0 (0.0%) | 0 (0.0%) | 1 (100.0%) | 1 | N.A. |
| Blocked amino end (Cys) | 0 (0.0%) | 0 (0.0%) | 0 (0.0%) | 1 (100.0%) | 1 | N.A. |
| Blocked amino end (Glu) | 0 (0.0%) | 0 (0.0%) | 0 (0.0%) | 1 (100.0%) | 1 | N.A. |
| Blocked amino end (Ile) | 0 (0.0%) | 0 (0.0%) | 0 (0.0%) | 1 (100.0%) | 1 | N.A. |
| Blocked amino end (Leu) | 0 (0.0%) | 0 (0.0%) | 0 (0.0%) | 1 (100.0%) | 1 | N.A. |
| Blocked amino end (Val) | 0 (0.0%) | 0 (0.0%) | 0 (0.0%) | 1 (100.0%) | 1 | N.A. |
| Blocked carboxyl end (Arg) | 0 (0.0%) | 0 (0.0%) | 0 (0.0%) | 1 (100.0%) | 1 | N.A. |
| Blocked carboxyl end (His) | 0 (0.0%) | 0 (0.0%) | 0 (0.0%) | 1 (100.0%) | 1 | N.A. |
| Isoleucine derivative | 0 (0.0%) | 0 (0.0%) | 0 (0.0%) | 1 (100.0%) | 1 | N.A. |
| Methionine derivative | 0 (0.0%) | 0 (0.0%) | 0 (0.0%) | 1 (100.0%) | 1 | N.A. |
| Tryptophan derivative | 0 (0.0%) | 0 (0.0%) | 0 (0.0%) | 1 (100.0%) | 1 | N.A. |
| N-acetylalanine | 3 (0.1%) | 844 (28.2%) | 7 (0.2%) | 2137 (71.4%) | 2991 | N-terminal |
| N-acetylmethionine | 4 (0.2%) | 655 (35.1%) | 8 (0.4%) | 1201 (64.3%) | 1868 | N-terminal |
| N-acetylserine | 3 (0.2%) | 693 (39.0%) | 12 (0.7%) | 1071 (60.2%) | 1779 | N-terminal |
| Pyrrolidone carboxylic acid | 5 (0.3%) | 343 (21.1%) | 1 (0.1%) | 1279 (78.6%) | 1628 | N-terminal |
| Pyruvic acid (Ser) | 0 (0.0%) | 0 (0.0%) | 0 (0.0%) | 1210 (100.0%) | 1210 | N-terminal |
| N-acetylthreonine | 0 (0.0%) | 72 (11.1%) | 1 (0.2%) | 577 (88.8%) | 650 | N-terminal |
| N-acetylproline | 0 (0.0%) | 68 (21.6%) | 0 (0.0%) | 247 (78.4%) | 315 | N-terminal |
| N-acetylvaline | 1 (0.5%) | 6 (3.0%) | 0 (0.0%) | 196 (96.6%) | 203 | N-terminal |
| N-formylmethionine | 0 (0.0%) | 5 (2.9%) | 0 (0.0%) | 169 (97.1%) | 174 | N-terminal |
| N-acetylglycine | 1 (0.6%) | 26 (16.0%) | 0 (0.0%) | 136 (83.4%) | 163 | N-terminal |
| N-acetylaspartate | 0 (0.0%) | 3 (2.4%) | 0 (0.0%) | 122 (97.6%) | 125 | N-terminal |
| N-methylphenylalanine | 0 (0.0%) | 0 (0.0%) | 0 (0.0%) | 74 (100.0%) | 74 | N-terminal |
| N2-acetylarginine | 0 (0.0%) | 10 (17.9%) | 0 (0.0%) | 46 (82.1%) | 56 | N-terminal |
| N,N,N-trimethylalanine | 0 (0.0%) | 35 (79.5%) | 0 (0.0%) | 9 (20.5%) | 44 | N-terminal |
| N-acetylcysteine | 0 (0.0%) | 2 (5.1%) | 0 (0.0%) | 37 (94.9%) | 39 | N-terminal |
| N-acetylglutamate | 0 (0.0%) | 3 (10.0%) | 0 (0.0%) | 27 (90.0%) | 30 | N-terminal |
| N-methylalanine | 1 (6.7%) | 4 (26.7%) | 0 (0.0%) | 10 (66.7%) | 15 | N-terminal |
| N,N-dimethylproline | 0 (0.0%) | 13 (86.7%) | 0 (0.0%) | 2 (13.3%) | 15 | N-terminal |
| N-methylmethionine | 0 (0.0%) | 1 (7.7%) | 0 (0.0%) | 12 (92.3%) | 13 | N-terminal |
| N-methylproline | 0 (0.0%) | 11 (100.0%) | 0 (0.0%) | 0 (0.0%) | 11 | N-terminal |
| 2-oxobutanoic acid | 0 (0.0%) | 2 (33.3%) | 0 (0.0%) | 4 (66.7%) | 6 | N-terminal |
| N,N-dimethylalanine | 0 (0.0%) | 4 (80.0%) | 0 (0.0%) | 1 (20.0%) | 5 | N-terminal |
| Pyrrolidone carboxylic acid (Glu) | 0 (0.0%) | 1 (25.0%) | 0 (0.0%) | 3 (75.0%) | 4 | N-terminal |
| Pyruvic acid (Cys) | 0 (0.0%) | 0 (0.0%) | 0 (0.0%) | 4 (100.0%) | 4 | N-terminal |
| 3-phenyllactic acid | 0 (0.0%) | 1 (33.3%) | 0 (0.0%) | 2 (66.7%) | 3 | N-terminal |
| N-methylleucine | 0 (0.0%) | 0 (0.0%) | 0 (0.0%) | 3 (100.0%) | 3 | N-terminal |
| N,N,N-trimethylserine | 0 (0.0%) | 1 (50.0%) | 1 (50.0%) | 0 (0.0%) | 2 | N-terminal |
| Lactic acid | 0 (0.0%) | 0 (0.0%) | 0 (0.0%) | 2 (100.0%) | 2 | N-terminal |
| N2,N2-dimethylarginine | 0 (0.0%) | 0 (0.0%) | 0 (0.0%) | 2 (100.0%) | 2 | N-terminal |
| N-acetyltyrosine | 0 (0.0%) | 0 (0.0%) | 0 (0.0%) | 2 (100.0%) | 2 | N-terminal |
| N-methyltyrosine | 0 (0.0%) | 0 (0.0%) | 0 (0.0%) | 2 (100.0%) | 2 | N-terminal |
| N,N-dimethylserine | 0 (0.0%) | 0 (0.0%) | 1 (100.0%) | 0 (0.0%) | 1 | N-terminal |
| N-methylserine | 0 (0.0%) | 0 (0.0%) | 1 (100.0%) | 0 (0.0%) | 1 | N-terminal |
| N,N-dimethylleucine | 0 (0.0%) | 0 (0.0%) | 0 (0.0%) | 1 (100.0%) | 1 | N-terminal |
| N2-succinyltryptophan | 0 (0.0%) | 0 (0.0%) | 0 (0.0%) | 1 (100.0%) | 1 | N-terminal |
| N-carbamoylalanine | 0 (0.0%) | 1 (100.0%) | 0 (0.0%) | 0 (0.0%) | 1 | N-terminal |
| N-D-glucuronoyl glycine | 0 (0.0%) | 1 (100.0%) | 0 (0.0%) | 0 (0.0%) | 1 | N-terminal |
| N-formylglycine | 0 (0.0%) | 0 (0.0%) | 0 (0.0%) | 1 (100.0%) | 1 | N-terminal |
| N-methylisoleucine | 0 (0.0%) | 0 (0.0%) | 0 (0.0%) | 1 (100.0%) | 1 | N-terminal |
| N-pyruvate 2-iminyl-cysteine | 0 (0.0%) | 0 (0.0%) | 0 (0.0%) | 1 (100.0%) | 1 | N-terminal |
| N-pyruvate 2-iminyl-valine | 0 (0.0%) | 0 (0.0%) | 0 (0.0%) | 1 (100.0%) | 1 | N-terminal |
| 2,3-didehydroalanine (Ser) | 0 (0.0%) | 0 (0.0%) | 0 (0.0%) | 292 (100.0%) | 292 | Protein core |
| 1-thioglycine | 0 (0.0%) | 0 (0.0%) | 0 (0.0%) | 86 (100.0%) | 86 | Protein core |
| 2,3-didehydroalanine (Cys) | 0 (0.0%) | 0 (0.0%) | 0 (0.0%) | 35 (100.0%) | 35 | Protein core |
| 2,3-didehydrobutyrine | 0 (0.0%) | 1 (3.6%) | 0 (0.0%) | 27 (96.4%) | 28 | Protein core |
| (Z)-2,3-didehydrobutyrine | 0 (0.0%) | 0 (0.0%) | 0 (0.0%) | 26 (100.0%) | 26 | Protein core |
| D-alanine (Ala) | 0 (0.0%) | 21 (95.5%) | 0 (0.0%) | 1 (4.5%) | 22 | Protein core |
| D-phenylalanine | 0 (0.0%) | 0 (0.0%) | 0 (0.0%) | 18 (100.0%) | 18 | Protein core |
| D-tryptophan | 0 (0.0%) | 0 (0.0%) | 0 (0.0%) | 13 (100.0%) | 13 | Protein core |
| D-leucine | 0 (0.0%) | 1 (10.0%) | 0 (0.0%) | 9 (90.0%) | 10 | Protein core |
| 2,3-didehydrotyrosine | 0 (0.0%) | 0 (0.0%) | 0 (0.0%) | 8 (100.0%) | 8 | Protein core |
| D-methionine | 0 (0.0%) | 2 (28.6%) | 0 (0.0%) | 5 (71.4%) | 7 | Protein core |
| D-allo-isoleucine | 0 (0.0%) | 1 (16.7%) | 0 (0.0%) | 5 (83.3%) | 6 | Protein core |
| (E)-2,3-didehydrobutyrine | 0 (0.0%) | 0 (0.0%) | 0 (0.0%) | 5 (100.0%) | 5 | Protein core |
| (E)-2,3-didehydrotyrosine | 0 (0.0%) | 0 (0.0%) | 0 (0.0%) | 3 (100.0%) | 3 | Protein core |
| (Z)-2,3-didehydrotyrosine | 0 (0.0%) | 0 (0.0%) | 0 (0.0%) | 3 (100.0%) | 3 | Protein core |
| 3'-nitrotyrosine | 0 (0.0%) | 0 (0.0%) | 0 (0.0%) | 3 (100.0%) | 3 | Protein core |
| Aspartyl isopeptide (Asn) | 0 (0.0%) | 0 (0.0%) | 0 (0.0%) | 3 (100.0%) | 3 | Protein core |
| D-alanine (Ser) | 0 (0.0%) | 0 (0.0%) | 0 (0.0%) | 3 (100.0%) | 3 | Protein core |
| D-4-hydroxyvaline | 0 (0.0%) | 0 (0.0%) | 0 (0.0%) | 2 (100.0%) | 2 | Protein core |
| D-serine (Ser) | 0 (0.0%) | 1 (50.0%) | 0 (0.0%) | 1 (50.0%) | 2 | Protein core |
| D-valine | 0 (0.0%) | 0 (0.0%) | 0 (0.0%) | 2 (100.0%) | 2 | Protein core |
| L-allo-isoleucine | 0 (0.0%) | 0 (0.0%) | 0 (0.0%) | 2 (100.0%) | 2 | Protein core |
| Aspartyl isopeptide (Asp) | 1 (100.0%) | 0 (0.0%) | 0 (0.0%) | 0 (0.0%) | 1 | Protein core |
| 2-methylglutamine | 0 (0.0%) | 0 (0.0%) | 0 (0.0%) | 1 (100.0%) | 1 | Protein core |
| D-asparagine | 0 (0.0%) | 1 (100.0%) | 0 (0.0%) | 0 (0.0%) | 1 | Protein core |
| D-serine (Cys) | 0 (0.0%) | 0 (0.0%) | 0 (0.0%) | 1 (100.0%) | 1 | Protein core |
| D-threonine | 0 (0.0%) | 0 (0.0%) | 0 (0.0%) | 1 (100.0%) | 1 | Protein core |

**Supplementary Figure 1:** Structural overlap of 11 chains from the following PDB structures: 3VVW:B, 3VH3:B, 3RUI:B, 2ZJD:A, 3D32:A, 1EO6:A, 2K6Q:A, 2LI5:A, 2KQ7:A, 2KWC:A, 1GNU:A. These protein structures were the result of a BLAST search (E-value< 0.001) for UniProtKB/Swiss-Prot sequences whose C-terminal glycine was annotated to be chemically modified (phosphatidylethanolamine amidated glycine). The C-terminal glycine is displayed in ball representation with an atom-dependent colored scheme.

**Supplementary Figure 2:** Structural superposition of 2NGR:A, 2DFK:B, 1A4R:A and 4F38:A. The structure of geranylgeranylated RhoA (4F38:A) was elucidated with its lipid anchor (colored yellow). Sequence numbering in 4F48:A is shifted by 2 residues when compared to the other 3 structures. These were not solved in the presence of a lipid anchor. Instead, the intramolecular disulfide bond between Cys105 and Cys188 is present (colored according to element color and red, respectively).

**Supplementary Figure 3:** PDB structure 4DM9 [48] of ubiquitin C-terminal hydrolase L1 (UCHL1). Residue 220 (target cysteine to farnesylation) is highlighted with ball and stick representation. A region colored in green, formed by a small β-strand/α helix motif, flanked by Gly24 and Pro45, is hypothesized to swing away in order to allow access of the modifying enzyme to the C-terminal of the protein. UCHL1 has been reported to exist in a membrane associated form, where its C-terminal cysteine undergoes farnesylation [49].

**Supplementary Figure 4:** Crystal structure of *Xenopus laevis* Wnt8 (in orange) in complex with the cysteine-rich domain of Frizzled 8 (in gray), with the modified serine 187 in red together with the palmitoleic acid lipid group in yellow [58].

**Supplementary Figure 5:** Electron cryo-microscopy of Chikungunya virus displaying the glycoprotein E1 in red and its transmembrane helix in blue. The associated position of the modified cysteine mapped to this structure is labeled to cysteine 433 and shown in ball and stick.

**Supplementary Figure 6:** Blast alignment of human P-selectin to the PDB structure of apolipoprotein-H with the modified cysteine 807 highlighted in red. The respective cysteine 288 found in the structure of the apolipoprotein-H 1C1Z is highlighted in ball and stick to form a disulfide bond to cysteine 326.

**Supplementary Figure 7:** Structures of two non-specific lipid-transfer proteins from wheat (left) and barley (right). The modified annotated residue aspartate is shown in ball and stick adopting different rotamers between the two structures.

**Supplementary Figure 8:** Examples of significant structural hits of C-terminal PTMs with positive score, but with low accessibility. Chains where PTM occurs are colored yellow. (a) Alpha-aminoadipate/glutamate carrier protein LysW, related to the annotation of PTMs 5-glutamyl 2-aminoadipic acid, 5-glutamyl N2-lysine, 5-glutamyl N2-arginine and 5-glutamyl N2-glutamate. (b) Mycobacterium protein involved in cysteine biosynthesis related to the annotation of CysO-cysteine adduct. (c) and (d) are representative structures for the complex between PP2A-specific methylesterases and the catalytic subunit PP2A, related to the annotation of Leucine methyl ester. (e) and (f) are representative structures for complexes with a sulfur carrier subunit that has been related to the glycyl adenylate PTM annotation. C-terminal thiocarboxylation of these residues was shown to be a necessary step for the active site formation.

**Supplementary Figure 9:** Number of PTM instances of (*a*) phosphoserine, (*b*) phosphothreonine, (*c*) phosphotyrosine and (*d*) phosphohistidine mapped to a protein structure according to the classification *STRUCTURE* (blue), *STRUCTURE-DIFF* (green), (*c*) *DISORDER* (yellow) or (*d*) *TRUE UNKNOWN* (red) as described in Methods. The additional light colored groups preceded by the affix *PD-* refers to the number of instances that, in addition to being mapped to one of the four descriptions, were predicted to be disordered by IUPred [29] using our benchmarked settings [30].

**Supplementary Figure 10:** Structures of elongation factor P (*a*) 3A5Z:B [43] and (*b*) 3TRE:A (Cheung *et al*., to be published). Residue 34 annotated to be modified (PTM N6-(3,6-diaminohexanoyl)-5-hydroxylysine) is displayed in ball and stick, colored according to its different elements. The accessibility of residue 34 in 3A5Z:B was calculated to be 0.2, while in 3TRE:A it was 1 (fully exposed). Clearly, the residue 34 in the structure 3A5Z:B is being hidden by the interaction of its putative lysyl-tRNA synthetase colored in blue.

**Supplementary Figure 11:** Structures of 1B33 and 1ON7 superimposed to highlight the methylated (red) and unmethylated (yellow) asparagine residues of 1B33 and 1ON7, respectively.

**Supplementary Figure 12:** Percentage of MOD_RES PTM sites that fulfilled the same requirements of those displayed in Fig. 5 of the paper, mapped to a DisProt region (red), predicted as disordered by IUPred (blue) or both (green) in regard to the total number of instances annotated (black curve). Others (gray) refer to all instances that did not fulfill any of the above. The average accessibility with its standard deviation calculated per PTM type is displayed with the mark (-). The calculated 3D structure score is shown in parenthesis for each PTM type labelled in the x-axis.

**Supplementary Figure 13:** Percentage of lipid PTM sites mapped to a DisProt region (red), predicted as disordered by IUPred (blue) or both (green) in regard to the total number of instances annotated (black curve). Others (gray) refer to all instances that did not fulfill any of the above. The average accessibility with its standard deviation calculated per PTM type is displayed with the mark (-). The calculated 3D structure score is shown in parenthesis for each PTM type labelled in the x-axis.
